# Childhood infections and autism spectrum disorders and/or intellectual disability: a register-based cohort study

**DOI:** 10.1186/s11689-022-09422-4

**Published:** 2022-02-13

**Authors:** Håkan Karlsson, Hugo Sjöqvist, Martin Brynge, Renee Gardner, Christina Dalman

**Affiliations:** 1 Department of Neuroscience, Karolinska Institutet, 171 77 Stockholm, Sweden; 2 Department of Global Public Health, Karolinska Institutet, 171 77 Stockholm, Sweden

**Keywords:** Childhood, Infection, Autism spectrum disorders, Intellectual disability, Risk

## Abstract

**Objective:**

To explore the associations between childhood infections and subsequent diagnoses of autism spectrum disorder (ASD), intellectual disability (ID), and their co-occurrence.

**Methods:**

The association between specialized care for any infection, defined by ICD-codes, and later ASD or ID was investigated in a register-based cohort of 556,732 individuals born 1987–2010, resident in Stockholm County, followed from birth to their 18th birthday or December 31, 2016. We considered as potential confounders children’s characteristics, family socioeconomic factors, obstetric complications, and parental histories of treatment for infection and psychiatric disorders in survival analyses with extended Cox regression models. Residual confounding by shared familial factors was addressed in sibling analyses using within-strata estimation in Cox regression models. Sensitivity analyses with the exclusion of congenital causes of ASD/ID and documented risk for infections were also performed.

**Results:**

Crude estimates indicated that infections during childhood were associated with later ASD and ID with the largest risks observed for diagnoses involving ID. Inclusion of covariates, exclusion of congenital causes of ASD/ID from the population, and sibling comparisons highlighted the potential for confounding by both heritable and non-heritable factors, though risks remained in all adjusted models. In adjusted sibling comparisons, excluding congenital causes, infections were associated with later “ASD without ID” (HR 1.24, 95%CI 1.15–1.33), “ASD with ID” (1.57, 1.35–1.82), and “ID without ASD” (2.01, 1.76–2.28). Risks associated with infections varied by age at exposure and by age at diagnosis of ASD/ID.

**Conclusions:**

Infections during childhood may contribute to a later diagnosis of ID and ASD.

**Supplementary Information:**

The online version contains supplementary material available at 10.1186/s11689-022-09422-4.

## Introduction

The etiology of autism spectrum disorders (ASD) remains elusive but appears to involve common, rare, and de novo genetic variants as well as environmental exposures [1]. The relative contributions of these plausibly causal factors appear to vary across diagnostic sub-groups based on co-occurring diagnoses [2–4]. Intellectual disability (ID, IQ <70) is diagnosed in around 1/3 of those diagnosed with ASD [5]. Individuals diagnosed with ASD with co-occurring ID are less likely to have a family history of ASD and other psychiatric diagnoses than individuals diagnosed with ASD without ID, indicating that ASD with ID is less heritable compared to ASD without ID [6–8]. De novo mutations, shared between ASD and ID, have been reported to play an important role for their co-occurrence, particularly among severely affected individuals [9, 10]. ASD is diagnosed throughout childhood and age at diagnosis appears to be determined by both internal (severity, sex) and external factors (parental educational level, access to care) [11]. While parents often report deviations from normal developmental trajectories at an early age, the pattern of onset varies substantially. It remains to be established if the childhood environment contributes to a trajectory towards ASD in vulnerable children [12, 13] Identification of modifiable early life environmental factors is key to the development of prevention strategies for ASD.

Infections that can invade the fetal and neonatal brain are established causes of life-long behavioral and intellectual disabilities [14, 15]. Infections during childhood have also been associated with psychiatric illness, including ID, later in life [16–20]. The extent to which some of these associations are causal or confounded by the genetic causes of ID or other psychiatric outcomes is currently not known. Recent reports associate deficits in both innate [21] and antibody-mediated [22] immunity at birth as well as variation in genes encoding proteins involved in immunity [23, 24] with ASD. Moreover, a genetic correlation between hospital care for infections and ASD has been reported [25]. Taken together, these observations suggest that infections during early childhood are potentially associated also with ASD. However, only a handful of studies, with equivocal results, have investigated the relationship between infections and the risk of later ASD [20, 26–30], and no epidemiological study to date has considered the potential confounding of any such association by factors shared between family members.

We here aimed to explore the associations between infections and subsequent diagnoses of ASD and ID and to understand whether any observed associations varied by their co-occurrence, age at infection, or age at first diagnosis.

## Methods

### Study population and general design

We defined a register-based cohort of 556,732 individuals born 1987–2010, resident in Stockholm County for ≥3 years [31]. Individuals not born in Sweden, adopted, not registered in the Medical Birth Register (MBR), or missing maternal data were excluded from the study (Fig. S1, available online).

Exposure, outcome, and covariate data were extracted from national and regional registers containing routinely collected health and sociodemographic data cross-linked via each resident’s unique national identification number [32]. The study was approved by the Stockholm regional ethics review board (DNR 2010/1185-31/5). Informed consent was not required to use anonymized register data.

### Diagnoses of ASD and ID

**Fig. 1:**
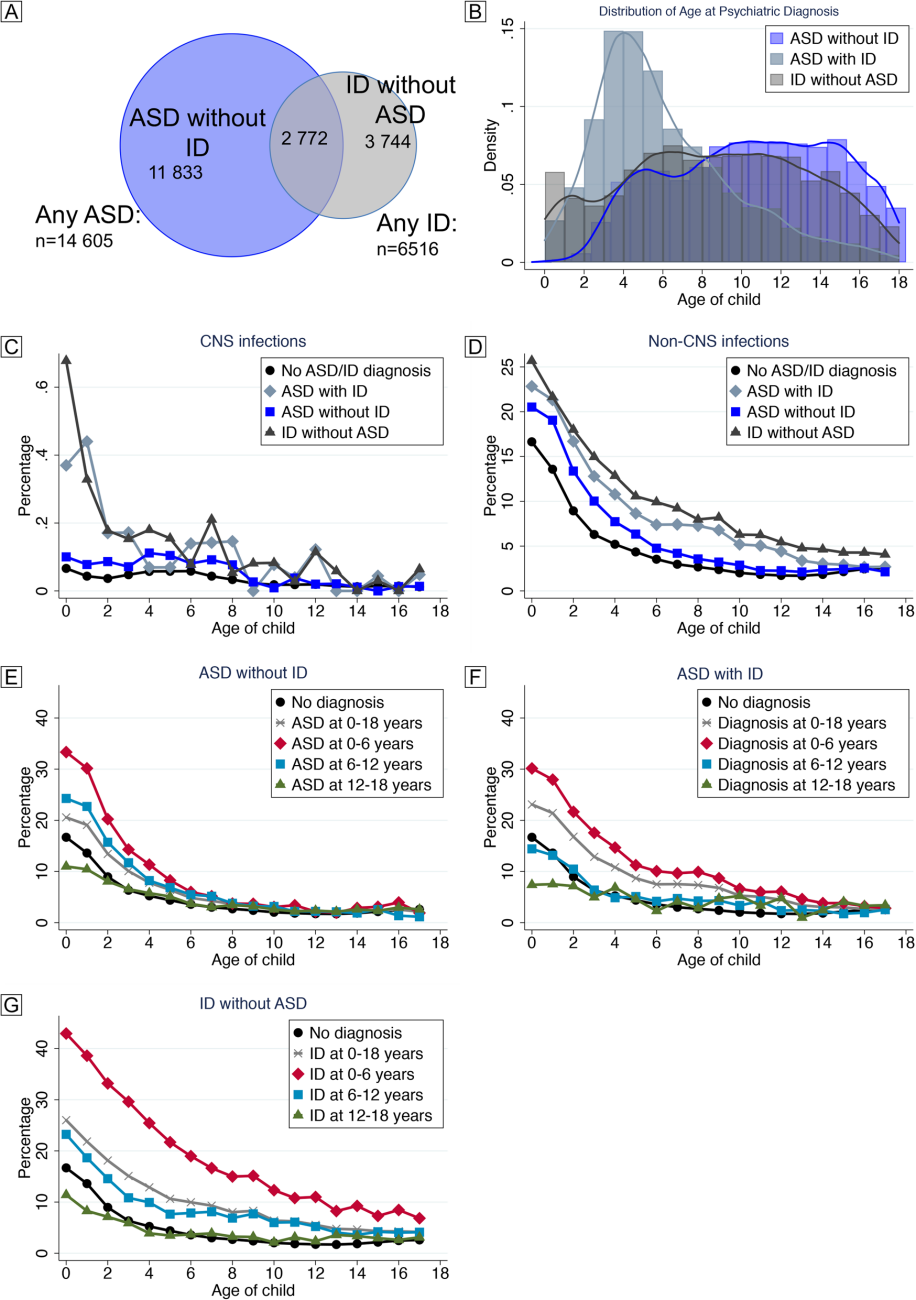
Description of outcomes and exposures in the study population. Individuals diagnosed with ASD and/or ID identified in the final study population (*n*=556,732) as of December 31, 2016 (**A**). Age at first diagnosis of ASD/ID for the mutually exclusive diagnostic groups (**B**). Incidence of CNS (**C**) and non-CNS infections (**D**) according to age among children unaffected by ASD/ID and among those diagnosed with “ASD without ID,” “ASD with ID,” “ID without ASD.” Note the different scales of the *y*-axes in **C** and **D**. Incidence of one or more infections according to age among children unaffected by ASD/ID and those diagnosed with “ASD without ID” (**E**), “ASD with ID” (**F**), and “ID without ASD” (**G**), throughout childhood and stratified by age at diagnosis of ASD/ID

Diagnostic outcomes as of December 31, 2016, were defined by validated procedures covering all inpatient and outpatient pathways to care and diagnosis in Stockholm County [31, 33] (ICD-9: ASD 299, ID 317-19, ICD-10: ASD F84, ID F70-79). We considered two overlapping diagnostic groups (individuals who received any diagnosis of ASD or any diagnosis of ID), as well as the three mutually exclusive diagnostic outcomes: ASD without co-occurring ID (“ASD without ID”), ASD with co-occurring ID (“ASD with ID”), and ID without co-occurring ASD (“ID without ASD”) (Fig. 1A).

### Assessment of childhood infections

All in- or outpatient specialized care diagnoses of infection (any infection), defined by ICD-8/9/10 codes as detailed previously [4, 34, 35], were identified in the National Patient Register (NPR). We omitted all codes “sequel” and “post.” Infections were also categorized according to timing (age at infection, see below) and site (CNS/non-CNS), see Table S1.

### Covariates

Covariates whose distribution varied by exposure to infection between birth and age 18 (Table 1) and that were associated with ASD or ID (Tables S2 and S3) were considered as potential confounders. Our register sources consisted of the Multigenerational Register (MR), the Longitudinal Integrated Database for Health Insurance and Labor Markets Studies (LISA), MBR, and NPR.

**Table 1:** Association between covariates and infection during childhood

		No infection	Any infection	***p*** value
***N***		274,137	282,595	
**Sex**	Male	137,874 (50.3%)	147,580 (52.2%)	<0.001
	Female	136,263 (49.7%)	135,015 (47.8%)	
**Birth order**	First	120,946 (44.1%)	130,474 (46.2%)	<0.001
	Second	100,348 (36.6%)	100,812 (35.7%)	
	≥Third	52,843 (19.3%)	51,309 (18.2%)	
**Season of birth**	Dec–Feb	63,102 (23.0%)	67,535 (23.9%)	<0.001
	Mar–May	76,236 (27.8%)	74,189 (26.3%)	
	Jun–Aug	71,724 (26.2%)	72,737 (25.7%)	
	Sep–Nov	63,075 (23.0%)	68,134 (24.1%)	
**GA**	Preterm	12,538 (4.6%)	18,647 (6.6%)	<0.001
	Term	259,245 (94.6%)	261,986 (92.7%)	
	Post term	1868 (0.7%)	1507 (0.5%)	
	Missing	486 (0.2%)	455 (0.2%)	
**Size for GA**	Small	5955 (2.2%)	7154 (2.5%)	<0.001
	Normal	252,060 (91.9%)	256,864 (90.9%)	
	Large	7756 (2.8%)	8866 (3.1%)	
	Missing	8366 (3.1%)	9711 (3.4%)	
**Cesarean delivery**	No	250,805 (91.5%)	250,794 (88.7%)	<0.001
	Yes	14,966 (5.5%)	22,090 (7.8%)	
	Missing	8366 (3.1%)	9711 (3.4%)	
**Low Apgar score (<7)**	No	268,435 (97.9%)	276,398 (97.8%)	<0.001
	Yes	2069 (0.8%)	3010 (1.1%)	
	Missing	3633 (1.3%)	3187 (1.1%)	
**Pre-eclampsia**	No	267,511 (97.6%)	274,246 (97.0%)	<0.001
	Yes	6626 (2.4%)	8349 (3.0%)	
**Maternal BMI**	Underweight	7742 (2.8%)	7358 (2.6%)	<0.001
	Normal weight	136,642 (49.8%)	145,232 (51.4%)	
	Overweight	35,625 (13.0%)	45,130 (16.0%)	
	Obese	10,994 (4.0%)	16,576 (5.9%)	
	Missing	83,134 (30.3%)	68,299 (24.2%)	
**Maternal age**	<25	39,398 (14.4%)	43,294 (15.3%)	<0.001
	25–29	81,387 (29.7%)	82,534 (29.2%)	
	30–34	95,776 (34.9%)	97,574 (34.5%)	
	35–39	47,716 (17.4%)	48,881 (17.3%)	
	>39	9860 (3.6%)	10,312 (3.6%)	
**Paternal age**	<25	18,213 (6.7%)	19,849 (7.1%)	<0.001
	25–29	59,360 (21.9%)	61,309 (21.9%)	
	30–34	91,088 (33.6%)	93,622 (33.5%)	
	35–39	63,265 (23.3%)	64,659 (23.1%)	
	>39	39,336 (14.5%)	40,240 (14.4%)	
**Mother’s region of origin**	Sweden	205,870 (75.1%)	202,203 (71.6%)	<0.001
	Nordic	8595 (3.1%)	6840 (2.4%)	
	West Eu and NA	12,282 (4.5%)	12,224 (4.3%)	
	Africa	11,013 (4.0%)	14,602 (5.2%)	
	Asia and Oceania	5423 (2.0%)	6239 (2.2%)	
	Middle-East	19,195 (7.0%)	27,227 (9.6%)	
	East Eu and Russia	3640 (1.3%)	3822 (1.4%)	
	South America	5230 (1.9%)	6502 (2.3%)	
	Unknown/missing	2889 (1.1%)	2936 (1.0%)	
**Father’s region of origin**	Sweden	209,664 (76.5%)	208,152 (73.7%)	<0.001
	Nordic	11,306 (4.1%)	8510 (3.0%)	
	West Eu and NA	8137 (3.0%)	8203 (2.9%)	
	Africa	9342 (3.4%)	12,877 (4.6%)	
	Asia and Oceania	8740 (3.2%)	8820 (3.1%)	
	Middle-East	16,377 (6.0%)	23,586 (8.3%)	
	East Eu and Russia	5821 (2.1%)	6307 (2.2%)	
	South America	4738 (1.7%)	6135 (2.2%)	
	Unknown/missing	12 (<1%)	5 (<1%)	
**Maternal psychiatric diagnosis**	No	165,792 (60.5%)	148,803 (52.7%)	<0.001
	Yes	108,345 (39.5%)	133,792 (47.3%)	
**Paternal psychiatric diagnosis**	No	203,417 (74.2%)	200,609 (71.0%)	<0.001
	Yes	70720 (25.8%)	81,986 (29.0%)	
**Maternal infection**	No	129,656 (47.3%)	92,021 (32.6%)	<0.001
	Yes	144,481 (52.7%)	190,574 (67.4%)	
**Paternal infection**	No	171,631 (62.6%)	150,049 (53.1%)	<0.001
	Yes	102,506 (37.4%)	132,546 (46.9%)	
**Family income**	Low (0–20%)	39,139 (14.3%)	40,758 (14.4%)	<0.001
	20–40%	55,086 (20.1%)	62,326 (22.1%)	
	40–60%	58,526 (21.3%)	62,289 (22.0%)	
	60–80%	59,933 (21.9%)	59,787 (21.2%)	
	High (80–100%)	61,126 (22.3%)	57,027 (20.2%)	
	Missing	327 (0.1%)	408 (0.1%)	
**Parental education**	<10 years	15,585 (5.7%)	18,908 (6.7%)	<0.001
	10–12	103,202 (37.6%)	109,598 (38.8%)	
	>12	154,608 (56.4%)	153,380 (54.3%)	
	Missing	742 (0.3%)	709 (0.3%)	
**ASD**	No ASD	268,334 (97.9%)	273,793 (96.9%)	<0.001
	0–<6 years	1132 (0.4%)	2572 (0.9%)	
	6–<12 years	2257 (0.8%)	3588 (1.3%)	
	12–18 years	2414 (0.9%)	2642 (0.9%)	
**ID**	No ID	272,086 (99.3%)	278,130 (98.4%)	<0.001
	0–<6 years	524 (0.2%)	1886 (0.7%)	
	6–<12 years	908 (0.3%)	1802 (0.6%)	
	12–18 years	619 (0.2%)	777 (0.3%)	

*GA* gestational age, *NA* North America, *Eu* Europe, *ASD* autism spectrum disorder, *ID* intellectual disability

We included the following factors: sex (MBR), birth order (MBR), birth season (MBR), gestational age at birth (MBR), size for gestational age (MBR), birth by cesarean section (MBR), and Apgar score 5 min after birth (MBR). We also included the pregnancy factors: pre-eclampsia (MBR) and maternal BMI at the first visit to antenatal care (categorized according to WHO standards [36], MBR). We also included the following parental factors: age at birth of the index child (MR), region of origin (MR), psychiatric history (defined as ICD10 F-chapter diagnosis, ICD9 290-315, or ICD8 290-315, NPR), and any infection diagnosis before birth of the index child (NPR). Finally, we included the following socioeconomic factors: family income at birth (quintiles of family income adjusted by inflation and size of family, recorded at the birth of the index person; LISA) and highest parental education at birth (LISA).

### Statistical analyses

We employed survival analysis with the extended Cox regression model using within-model stratification to account for differences by the children’s year of birth. Age of the child was used as the underlying timescale, beginning at birth. Individuals were followed until death, emigration from Stockholm, ASD/ID diagnosis, their 18th birthday, or end of study. For joint diagnosis (those individuals diagnosed with both ASD and ID), the date of the first diagnosis was used to calculate the person-time contributed to the study, though follow-up for ascertainment of the co-diagnosis continued until the end of study-time or an otherwise censoring event (such that individuals diagnosed with ID were not censored in terms of further follow-up for ASD, and vice versa). The infection variables were treated as time-dependent exposures, in which the individual could have had an infection from birth up to 18 years of age, given that the child was not diagnosed with an outcome of interest or otherwise censored. To account for the possible non-proportional risk of the outcomes over follow-up time, we considered both cumulative time, wherein the exposure to infection could occur from birth up to the 18th birthday, and stratified according to age at infection; 0–<1 year, 1–<3, 3–<6, 6–<12, and 12–<18. To test for differences in risk related to age at infection, we used the Wald test to evaluate the beta coefficients for each stratum of age at exposure in the cumulative models. To assess if the associations varied by age at outcome, we also stratified the outcome according to age at first diagnosis of ASD and/or ID: 0–<6, 6–<12, and 12–<18 years. To explore if sex modulated any potential association, fully adjusted analyses of the association between infections and ASD/ID were repeated for males and females, separately.

To explore potential confounding by factors (genes and environment) not accounted for by adjustment for covariates, we performed sibling analyses (siblings share their early environment and on average 50% of their DNA) using within-strata estimation in Cox regression models, adjusted for factors that (can) differ between siblings, sex, parity, birth season, GA at birth and cesarean section in which we discarded all only-child observations (*n*=131,335). Data management was done in SAS 9.4 and data analyses in Stata/MP 15.1.

### Sensitivity analyses

Individuals with rare chromosomal abnormalities are at increased risk for both ASD/ID and repeated specialized care for infections during infancy and childhood [37, 38]. To address if such individuals were driving potential associations observed, we repeated the population and sibling comparison analyses after excluding individuals affected by a study outcome who were also diagnosed with congenital deformation or chromosomal abnormalities (ICD-9: 758, 759F, 237H, ICD-10: Q85.0, Q85.2, Q90-99), and disorders of amino acid metabolism (ICD-9: 270, ICD-10: E70-72), as detailed previously [33].

## Results

### Description of the study population

Of the 556,732 children included in our study (Fig. S1, available online), 282,595 (50.8%) had at least one registered infection before age 18. All investigated covariates differed significantly (*p*<0.001) between those who received specialized care for infection and those who did not (Table 1). Among those who were not diagnosed with an infection during childhood, 5803 (2.1%) were diagnosed with ASD and 2051 (0.7%) with ID. Among those diagnosed with one or more infection, 8802 (3.1%) were diagnosed with ASD and 4465 (1.6%) with ID (Table 1). As compared to “ASD without ID” or “ID without ASD,” “ASD with ID” was diagnosed at an earlier age (Fig. 1B). CNS infections were rare as compared to non-CNS infections. Both types of infections were overrepresented in individuals with the investigated outcomes, particularly those involving ID (Fig. 1C, D). Infections were more common during the first few years of childhood in all diagnostic groups (Fig. 1E–G), particularly among those with an early diagnosis of “ID without ASD” or “ASD with ID,” as compared to those without a diagnosis of ASD/ID.

All included covariates were associated with ASD and age at ASD diagnosis. For example, ASD was more common among males and first-born children, particularly among those diagnosed before age 6 (Table S2, available online). Similarly, obstetric complications, such as pre-term birth and cesarean section were associated with early diagnosis whereas parental psychiatric history was more common among cases diagnosed after age 12. Similar observations were made for ID (Table S3, available online).

### Main analyses

#### Overlapping diagnostic outcomes

**Fig. 2:**
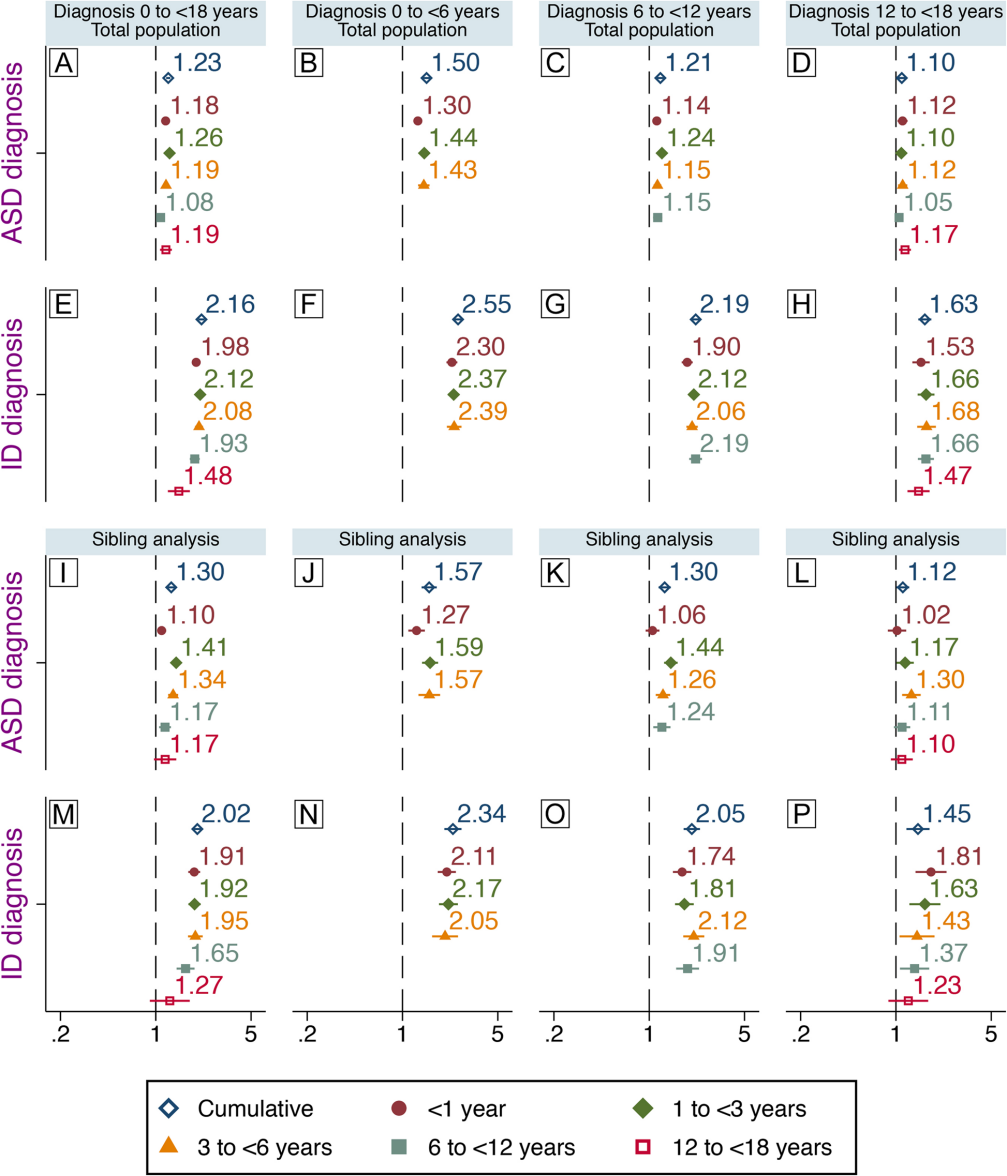
Infections during childhood and later diagnosis of ASD or ID. Associations between specialized care for infections during childhood and later, non-mutually exclusive, diagnosis of ASD and ID. Associations between exposure at different age intervals and diagnoses at different age intervals are also shown. Comparison between unrelated individuals in the general population are shown (**A**–**H**) and comparisons between full biological siblings (**I**–**P**). Hazard ratios presented here are from fully adjusted models. Population-based estimates (**A**–**H**) are adjusted for sex, parity, maternal body mass index, pre-eclampsia, parental age, education, income, region of origin, histories of psychiatric illness and infections, season of birth, gestational age at birth, size for gestational age, cesarean section, Apgar score. Estimates from the sibling analyses (**I**–**P**) are adjusted for sex, parity, gestational age at birth, and cesarean section

Inclusion of covariates in our fully adjusted models (Fig. 2) attenuated risk estimates compared to crude models (Fig. S2, available online) but did not change the overall findings. One-by-one, individual co-variates variably, but minimally, modulated the association between childhood infections and later diagnosis of ASD or ID (Fig. S3).

In fully adjusted models, infections during childhood (0–<18 years) were associated with later ASD (HR 1.23, 95%CI 1.19–1.28) and ID diagnoses (HR 2.16, 95%CI 2.05–2.28). Infections at all age intervals except 6-<12 were associated with later ASD, with the strongest association observed for infections in the age interval 1–<3 (HR 1.26, 95%CI 1.21–1.31) Figure 2A. Infections at all ages were associated with a later ID diagnosis, with the strongest association observed for infections during age 1–<3 (HR 2.12, 95%CI 2.00–2.24) (Fig. 2E).

Infections were associated with ASD diagnosed at age 0–<6 (HR 1.50, 95%CI 1.40–1.61, Fig. 2B) and 6–<12 (HR 1.21, 95%CI 1.14–1.28, Fig. 2C), but not 12–<18 (Fig. 2D). Infections were associated with ID diagnosed throughout the follow-up (Fig. 2F–H), with the strongest association during ages 0–<6 (HR 2.55, 95%CI 2.33–2.80, Fig. 2F). Stratified analyses indicated no major modulating effect of sex although we observed slightly stronger associations between infections and both ASD and ID among females than among males (Fig. S4).

Sibling comparison models resulted in associations generally comparable to those observed in the total population analysis, see Fig. 2I–P. Notably, infections during age 1–<3 exhibited a stronger association with ASD diagnosed before age 12 (Fig. 2J, K). Only infections 6–<12 were associated with ASD diagnosed at 12–<18 (Fig. 2L). Infections at 12–<18 were not associated with ASD or ID.

### Mutually exclusive outcomes

**Fig. 3:**
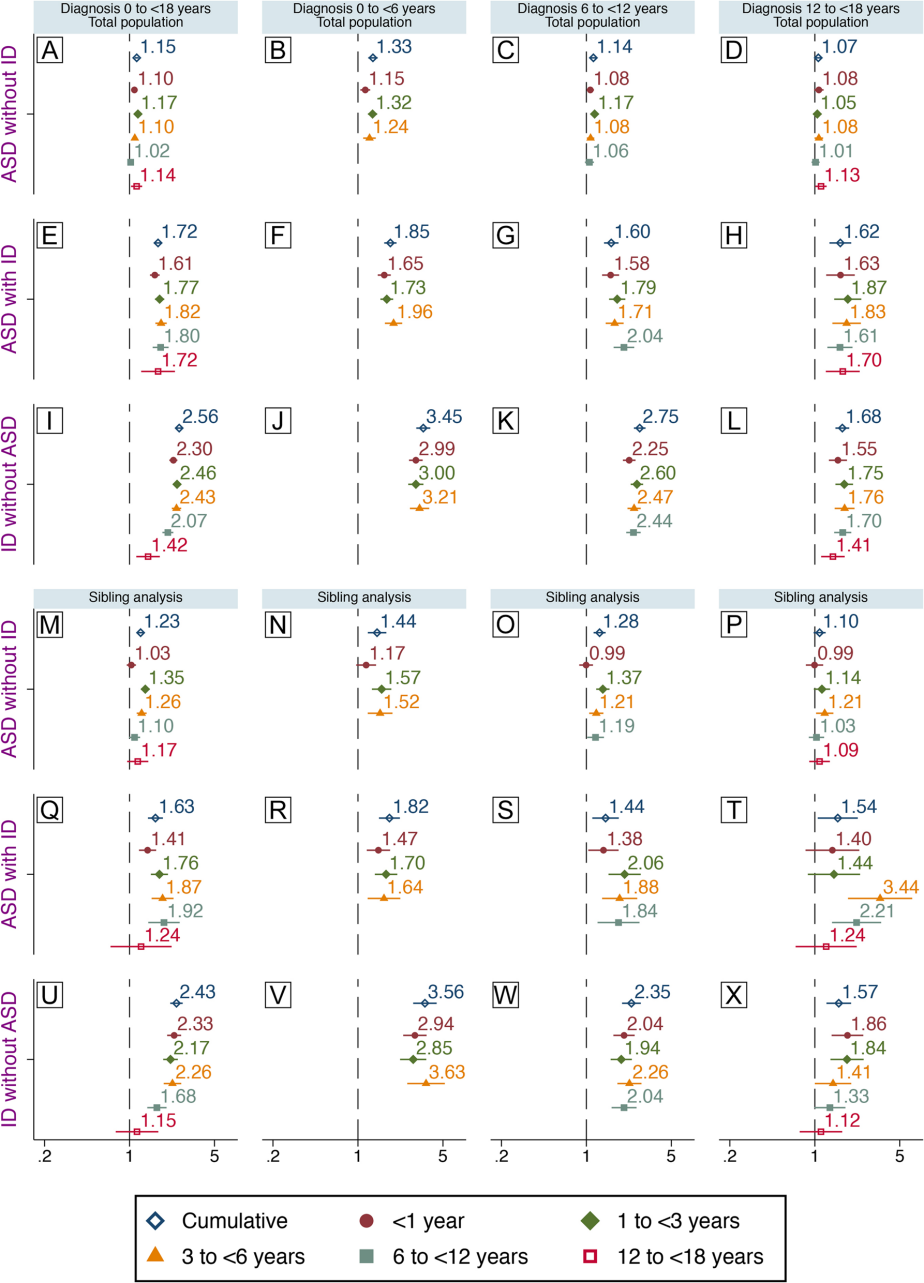
Infections during childhood and mutually exclusive diagnoses. Associations between specialized care for infections during childhood and later diagnosis of “ASD without ID,” “ASD with ID,” or “ID without ASD.” Associations between exposure at different age intervals and diagnoses at different age intervals are also shown. Comparisons between unrelated individuals in the general population (**A**–**L**) and between full biological siblings (**M**–**X**) are shown. Hazard ratios presented here are from fully adjusted models. Population-based estimates (**A**–**L**) are adjusted for sex, parity, maternal body mass index, pre-eclampsia, parental age, education, income, region of origin, histories of psychiatric illness and infections, season of birth, gestational age at birth, size for gestational age, cesarean section, Apgar score. Estimates from the sibling analyses (**M**–**X**) are adjusted for sex, parity, gestational age at birth, and cesarean section

Similar results regarding the mutually exclusive outcome groups were observed in crude (Fig. S5, available online) and adjusted models (Fig. 3). Infections during childhood were associated with the mutually exclusive diagnoses with the largest risk estimates observed for “ID without ASD” (HR 2.56, 95%CI 2.39–2.75, Fig. 3I) and the smallest for “ASD without ID” (HR 1.15, 95%CI 1.10–1.19, Fig. 3A). Whereas infections at all ages were associated with “ID without ASD” and “ASD with ID,” only those at 1–<3 and 3–<6 were significantly associated with “ASD without ID.” Larger-point estimates were observed among those diagnosed early. Infections were not associated with “ASD without ID” diagnosed after age 12 (Fig. 3B–D, F–H, and J–L).

Sibling analyses resulted in slightly modified associations as compared to those observed in the general population, with an enhanced risk for “ASD without ID” (Fig. 3M–P) and mostly attenuated risks for “ASD with ID” (Fig. 3Q–T) or “ID without ASD” (Fig. 3U–Y). Associations with infections at 12–<18 were attenuated towards unity for all outcomes. Higher risk estimates for “ASD without ID” appeared to be largely driven by infections in the age intervals 1–<3 and 3–<6 (Fig. 3M) among those diagnosed before age 6 (Fig. 3N) or at 6–<12 (Fig. 3O). Infections during the first year of life were associated with later “ASD with ID” (Fig. 3Q) with the largest risk estimate observed among those diagnosed before age 6 (Fig. 3R). Infections before age 3 were no longer associated with “ASD with ID” diagnosed at 12–<18, though elevated risks were observed for infections at 3–<6 and 6–<12 (HR 3.44, 95%CI 1.87–6.33, and HR 2.21, 95%CI 1.39–3.51), respectively, Fig. 3T). Infections during early childhood remained strongly associated with “ID without ASD” (Fig. 3U), particularly among those diagnosed before age 6 (Fig. 3V) or at 6–<12 (Fig. 3X), but also among those diagnosed at 12–<18 (Fig. 3Y).

### Sensitivity analyses

Exclusion of children diagnosed with congenital disorders from the total population (Fig. S6) and sibling (Fig. S7, available online) comparisons attenuated the associations observed between infections and the mutually exclusive diagnoses involving ID. In the sibling comparison, infections at 1–<3 and 3–<6 remained associated with “ASD without ID.” Infections before age 12 remained associated with both “ASD with ID” and “ID without ASD” throughout the follow-up. Among those diagnosed at 12–<18, only infections before age 3 were associated with “ID without ASD,” whereas infections at 3–<6 and 6–<12 were associated with “ASD with ID” (Fig. S7).

### CNS and non-CNS infections

**Fig. 4:**
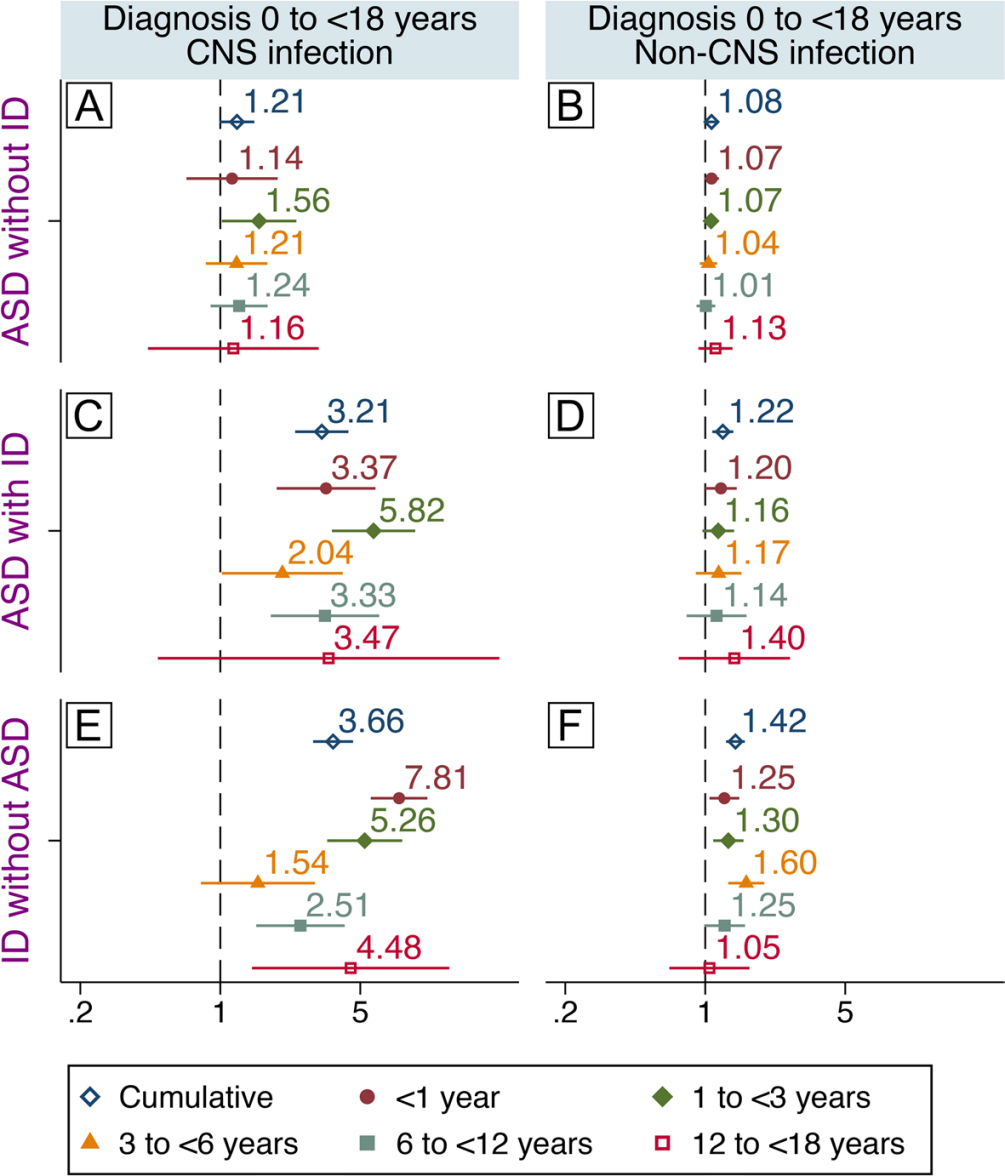
CNS/non-CNS infections and mutually exclusive diagnostic groups. Associations between specialized care for CNS infections (left) or non-CNS infections (right) during childhood and later diagnosis of “ASD without ID” (**A**, **B**), “ASD with ID” (**C**, **D**), or “ID without ASD” (**E**, **F**). Associations between exposure at different age intervals and later diagnoses are also shown. Hazard ratios are adjusted for sex, parity, maternal body mass index, pre-eclampsia, parental age, education, income, region of origin, histories of psychiatric illness and infections, season of birth, gestational age at birth, size for gestational age, cesarean section, and Apgar score

Larger risk estimates for all outcomes were generally observed for CNS infections than for non-CNS infections (Fig. 4), e.g., risk for “ID without ASD” was 3.66 (95% CI: 2.91–4.60, Fig. 4E) for CNS infections and 1.42 (95% CI: 1.27–1.58, Fig. 4F) for non-CNS infections.

## Discussion

In the present study, infections requiring specialized care between ages 1 and <6 were associated with the risk of being diagnosed with ‘ASD without ID’ before age 12. Infections before age 12 were associated with “ASD with ID” diagnosed throughout childhood. Similar observations were made for “ID without ASD” with even larger point estimates, particularly for infections during the first few years of childhood. We noted larger risks for all outcomes associated with CNS infections than for non-CNS infections, particularly for diagnoses involving ID. While our analyses highlighted the potential for confounding by both familial factors and factors not shared between family members, they also indicate that childhood infections potentially contribute to some cases of both ASD and ID.

Long-term follow-up studies of individuals with congenital infections have reported autism-like behaviors [39–41]. There are, however, few published studies of the potential association between infections overall and later ASD in the general population, none of which considered the potential differential effect on ASD with or without co-occurring ID or the potential for confounding by factors shared between family members.

Rosen et al. reported no association between inpatient, outpatient, emergency, or referral visits for infections registered within the Kaiser-Permanente health plan during the first 2 years of life and later ASD in a sample consisting of 500 cases and 2000 matched controls [28]. Atladottir et al., on the other hand, reported associations between hospitalization for infections during childhood and later ASD (HR 1.38) in a Danish population in 2010 [27], comparable to those observed here (HR 1.23). More recently, Sabourin and co-workers reported that individuals with ASD were more likely to have had an infection during the first 3 years of life than comparison individuals using a combination of neonatal records and care-giver interviews [29]. Our observations regarding CNS infections agree with those reported by Pedersen et al. [20], who found a weak association between CNS infections and ASD that disappeared after the exclusion of individuals with co-occurring ID, although we lacked the power to distinguish between effects of CNS and non-CNS infections.

With regard to the observed risk for ID, our results are in agreement with a number of clinical follow-up studies of neonates with congenital infections [15] and CNS-infections [42] of both viral and bacterial origin during both infancy and childhood [16–18, 43–45]. In a study employing Danish population registers [20], the authors explored a wide range of mental disorders in relation to previous CNS infections and reported a significant association (HR 3.29) between CNS infections and later ID, similar to those observed here for “ID without ASD” (HR 3.66) and for “ASD with ID”’ (HR 3.21).

Fewer studies have focused on cognitive function after childhood infections not involving the CNS. Kariuki et al. reported impairments in neurocognitive testing at age 7 in children who had been hospital treated for infections before age four [46]. Moreover, the Danish register study by Atladottir et al. reported an association between infections during childhood and later ID [27] of a magnitude similar to that observed here. Our current findings are also in line with previous studies where both CNS and non-CNS infections during early childhood were associated with lower scores on a mandatory cognitive test administered to conscripts, representative of the general male population [35, 47]. Taken together with these previous studies, our current observations, suggest that also infections without a recognized CNS involvement during childhood are associated with subsequent impaired cognitive function. Whether this association is causal is however not known. Genetic syndromes associated with ID such as Down syndrome, are associated with increased rates of severe infections during childhood presumably explained by anatomical and immune system abnormalities associated with trisomy 21 [37]. However, respiratory infections are significant causes of mortality and potentially also exacerbated cognitive impairments among exposed carriers [48].

Strengths of the study include the large, population-based cohort with prospectively collected data in a setting with universal access to health care with minimal risk for selection or recall bias. Though all clinical pathways to ASD/ID diagnosis are covered, we do not have access to individual assessments of cognitive function. Thus, children diagnosed as “ASD without ID” could experience impairments due to infection that do not meet criteria for ID. As well, children diagnosed with “ID without ASD” might experience some or many impairments related to ASD, but these aspects may be under-ascertained after a diagnosis of ID. We have previously reported that the more severe the ID diagnosis, the less likely the child is to receive a co-occurring ASD diagnosis [33]. Given the stronger relationship between childhood infections and risk of ID we observed and the much higher cumulative incidence of ASD in our population, misclassification of the co-occurring disorders might serve to slightly bias our estimates for the relationship between childhood infections and risk of ASD without ID away from the null, while biasing the estimates of “ID without ASD” towards the null. Despite the large study population, we had limited power to analyze specific infections. Moreover, potential mediation (and/or modification) of the observed relationships between childhood infections and ASD/ID by neurological, or other somatic conditions, are possible but were not investigated here.

Potential confounding was addressed in multiple ways. We adjusted our total-population estimates for a wide range of potentially confounding factors, though it should be noted that our estimates may still be influenced by residual confounding, especially by factors, such as maternal BMI where a relatively large proportion of observations were missing, and for factors that were unobserved or incompletely captured, such as genetic susceptibility to serious infections and neurodevelopmental disorders. We used sibling comparison models to account for residual confounding by factors shared between siblings (siblings share on average 50% of their DNA along with part of their environment) and adjusted for factors not necessarily shared between siblings (e.g., pre-term birth). Sensitivity analyses of both the general population and sibling comparisons indicated that the associations observed were not fully explained by higher rates of infections among children with chromosomal abnormalities or inborn errors of metabolism, although residual confounding, e.g., by undiagnosed rare or de novo variants, linked to both ASD and ID [10], cannot be excluded.

Age at diagnosis depends on a wide range of factors such as clinical practice, severity, and parental vigilance [11]. Results from studies using register-based data to investigate timing of an exposure in relation to the onset of a childhood disorder should therefore be interpreted cautiously. While infections during early childhood were more common in children diagnosed with ASD or ID than in children without such diagnoses, we also note that the group diagnosed with ID at a young age had an excess of infections throughout childhood. Such an excess of infections in the group of individuals diagnosed with ASD without ID, however, seemed to be limited to the first decade of life. While these groups contain individuals with underlying hereditary and non-hereditary causes of ASD/ID that also receive more specialized care for infections during childhood [49], this does not preclude infections from being casually linked to some cases of ID and ASD. A higher liability to infections would increase the risk of contracting a serious infection requiring specialized care during early childhood, arguably a period of development more sensitive to external insults than later parts of childhood as illustrated by the exacerbated language problems associated with recurrent ear infections, common among children with fragile X syndrome [50]. It should also be kept in mind that our exposure definition is broad and encompasses a range of infectious agents, each with a prevalence and consequences that are likely to vary by age [51], which complicates comparisons of effects of exposures at different ages.

A number of different, but not mutually exclusive, mechanisms can potentially explain our current observations. Targeting of the nervous system by infectious agents during periods of rapid growth and plasticity [52] is one plausible mechanism. Complications involving the CNS are, albeit rarely, observed for many different infectious agents, including those not normally associated with neuro-invasion, particularly in young individuals [51, 53, 54]. For example, respiratory viruses (e.g., influenza A virus, respiratory syncytial virus) are important causes of encephalitis in young children [55]. It is possible that for some registered infections, mild CNS involvement may have gone unnoticed or undocumented, as has been reported for uncomplicated measles virus infections [56]. Moreover, low levels of several molecules involved in the innate immune response at birth and primary antibody deficits were recently associated with ASD risk [21, 22], supporting the notion of an increased vulnerability to infections in ASD. Such an increased vulnerability to infections is supported by observations of a genetic correlation between hospitalization for infection and ASD [25] and the reported links between immune genes and risk of both ASD and ID [23, 24]. Further studies are needed to investigate if deficits in immunity associated with ASD risk could contribute to more severe outcomes of childhood infections, such as ID, and potentially contribute to the high co-morbidity between these disorders [57].

Direct support for immune mediated influences on the developing CNS resulting in cognitive and behavioral abnormalities is provided by experimental studies. Various protocols mimicking viral or bacterial infections during certain stages of development produce persistent cognitive deficits and behavioral changes in adult animals reminiscent of those observed in individuals with ASD [58]. Moreover, transient treatment with interferon-γ during neuronal differentiation of human stem cells was reported to cause induction of ASD-associated genes [59]. While studies of individuals diagnosed with ASD support activation of the immune system in ASD, the pathogenetic role of such immune activity remains unclear [60–63]. Nevertheless, many of the pathways activated by infections have pleiotropic effects during brain development [64]. Thus, the association between infections during periods of rapid growth and plasticity of the brain and later diagnosis of ASD/ID may be explained by components of the immune system interfering with the normal trajectories of brain development.

Infections cause changes in the distribution of both macro- and micronutrients in the host, with the purpose of promoting the function of the immune system while limiting pathogen proliferation [65, 66]. Indeed, febrile episodes during childhood have been associated with reduced adult height, independent of genetic background and nutritional intake [67]. Brain development during the first years of life is characterized by rapid, sequential patterns of cellular differentiation, migration, and synapse formation during which the demand for macro- and micronutrients is high [68]. Diversion of macro- and micronutrients necessary to mount an adequate immune response to clear an infection may explain part of our observed association between infections during childhood and later ID/ASD [69, 70].

## Conclusions

Specialized care for infections during childhood is common among individuals diagnosed with ID and/or ASD. It may be that children who are later diagnosed with ID or ASD have a higher liability to severe infections that contribute to the associations observed here but infections are not causing ID or ASD. It may also be that such an increased liability leads to increased exposure to severe infections, during critical periods, which adversely affect neurodevelopment. Based on our analysis of the timing of infections and diagnoses of ASD and ID in this study, including careful consideration of potential confounding by shared familial factors, we suggest that infections during early childhood contribute to risk of a later diagnosis of ID, including ID co-occurring with ASD. Our observation that infections during early childhood exhibit weak but significant associations with “ASD without ID” requires replication in future studies. While we cannot exclude that the associations observed here are still explained by residual confounding, the evidence offered here warrants the identification of specific infections, their mechanisms of action, and individuals at risk.

## Supplementary Information

**Additional file 1: Figure S1.** Flow chart delineating the derivation of the populations included in the present study. **Figure S2.** Infections during childhood and later diagnosis of ASD or ID. Crude associations between specialized care for infections and later, non-mutually exclusive, diagnosis of ASD and ID. Associations between exposure and diagnoses at different age interval are also shown. Comparison between unrelated individuals in the general population are shown (A-H) and comparisons between full biological siblings (I-P). **Figure S3.** An exploration of the influence of the different potentially confounding factors on effect estimates for childhood infections on risk of the mutually non-exclusive diagnoses of ASD (left) and ID (right). We used survival analysis with the extended Cox regression model to examine the relationship between childhood infections and each outcome adjusting for each potential confounder individually and compared these results to crude (top) and fully adjusted models (bottom). The effect estimates (HR) for infections after each adjustment are presented along with bands representing the 95% confidence intervals. **Figure S4.** Sex-stratified analyses of the association between childhood infections and the later diagnosis of ASD or ID. Fully adjusted associations between specialized care for infections and later, non-mutually exclusive, diagnosis of ASD and ID among males (top row) and females (bottom row). Associations between exposure and diagnoses at different age interval are also shown. Only comparison between unrelated individuals in the general population are shown. **Figure S5.** Infections during childhood and mutually exclusive diagnoses. Crude associations between infections between birth and age 18 and later diagnosis of ‘ASD without ID’, ‘ASD with ID’ or ‘ID without ASD’. Associations between exposures and diagnoses at different ages are also shown. Comparisons between unrelated individuals in the general population (A-L) and between full biological siblings (M-X) are shown. **Figure S6.** Infections during childhood and mutually exclusive diagnoses-sensitivity analyses. Associations between infections between birth and age 18 and later diagnosis of ‘ASD without ID’, ‘ASD with ID’ or ‘ID without ASD’ in the total population following exclusion of individuals with congenital diagnoses and ASD/ID. Associations between exposures and diagnoses at different ages are also shown. Estimates are adjusted for sex, parity, maternal body mass index, pre-eclampsia, parental age, education, income, region of origin, histories of psychiatric illness and infections, season of birth, gestational age at birth, size for gestational age, cesarean section and Apgar score. **Figure S7.** Infections during childhood and mutually exclusive diagnoses-sensitivity analyses. Associations between infections between birth and age 18 and later diagnosis of ‘ASD without ID’, ‘ASD with ID’ or ‘ID without ASD’ among biological siblings following exclusion of individuals with congenital diagnoses and ASD/ID. Associations between exposures and diagnoses at different ages are also shown. Estimates from are adjusted for sex, parity, gestational age at birth and cesarean section.

**Additional file 2: Table S1.** ICD codes for infection diagnoses. **Table S2.** Association between covariates and ASD and by age at first diagnosis. **Table S3.** Association between covariates and ID and by age at first diagnosis.

## Data Availability

Data are available upon request from the indicated agencies

## References

[1] Gaugler T (2014). Most genetic risk for autism resides with common variation. Nat Genet.

[2] Grove J (2019). Identification of common genetic risk variants for autism spectrum disorder. Nat Genet.

[3] Iossifov I (2014). The contribution of de novo coding mutations to autism spectrum disorder. Nature..

[4] Lee BK (2015). Maternal hospitalization with infection during pregnancy and risk of autism spectrum disorders. Brain Behav Immun.

[5] Maenner MJ (2020). Prevalence of Autism Spectrum Disorder Among Children Aged 8 Years - Autism and Developmental Disabilities Monitoring Network, 11 Sites, United States, 2016. MMWR Surveill Summ.

[6] Robinson EB (2014). Autism spectrum disorder severity reflects the average contribution of de novo and familial influences. Proc Natl Acad Sci U S A.

[7] Xie S (2019). Family History of Mental and Neurological Disorders and Risk of Autism. JAMA Netw Open.

[8] Xie S (2020). The Familial Risk of Autism Spectrum Disorder with and without Intellectual Disability. Autism Res.

[9] Srivastava AK, Schwartz CE (2014). Intellectual disability and autism spectrum disorders: causal genes and molecular mechanisms. Neurosci Biobehav Rev.

[10] Vissers LE, Gilissen C, Veltman JA (2016). Genetic studies in intellectual disability and related disorders. Nat Rev Genet.

[11] Shulman C, Esler A, Morrier MJ, Rice CE (2020). Diagnosis of Autism Spectrum Disorder Across the Lifespan. Child Adolesc Psychiatr Clin N Am.

[12] Ozonoff S, Iosif AM (2019). Changing conceptualizations of regression: What prospective studies reveal about the onset of autism spectrum disorder. Neurosci Biobehav Rev.

[13] Stoner R (2014). Patches of disorganization in the neocortex of children with autism. N Engl J Med.

[14] Johnson RT (1972). Effects of viral infection on the developing nervous system. N Engl J Med.

[15] Ostrander B, Bale JF (2019). Congenital and perinatal infections. Handb Clin Neurol.

[16] Chamberlain RN (1983). A study of school children who had identified virus infections of the central nervous system during infancy. Child Care Health Dev.

[17] Chang LY (2007). Neurodevelopment and cognition in children after enterovirus 71 infection. N Engl J Med.

[18] Michaeli O (2014). Long-term motor and cognitive outcome of acute encephalitis. Pediatrics..

[19] Dalman C (2008). Infections in the CNS during childhood and the risk of subsequent psychotic illness: a cohort study of more than one million Swedish subjects. Am J Psychiatry.

[20] Pedersen EMJ (2020). Infections of the central nervous system as a risk factor for mental disorders and cognitive impairment: A nationwide register-based study. Brain Behav Immun.

[21] Gardner RM (2021). Neonatal Levels of Acute Phase Proteins and Risk of Autism Spectrum Disorder. Biol Psychiatry.

[22] Isung J (2020). Association of Primary Humoral Immunodeficiencies With Psychiatric Disorders and Suicidal Behavior and the Role of Autoimmune Diseases. JAMA Psychiatry.

[23] Nudel R (2019). Immunity and mental illness: findings from a Danish population-based immunogenetic study of seven psychiatric and neurodevelopmental disorders. Eur J Hum Genet.

[24] Torres AR (2016). Common Genetic Variants Found in HLA and KIR Immune Genes in Autism Spectrum Disorder. Front Neurosci.

[25] Nudel R (2019). A large-scale genomic investigation of susceptibility to infection and its association with mental disorders in the Danish population. Transl Psychiatry.

[26] Atladottir HO, Henriksen TB, Schendel DE, Parner ET (2012). Autism after infection, febrile episodes, and antibiotic use during pregnancy: an exploratory study. Pediatrics..

[27] Atladottir HO (2010). Association of hospitalization for infection in childhood with diagnosis of autism spectrum disorders: a Danish cohort study. Arch Pediatr Adolesc Med.

[28] Rosen NJ, Yoshida CK, Croen LA (2007). Infection in the first 2 years of life and autism spectrum disorders. Pediatrics..

[29] Sabourin KR (2019). Infections in children with autism spectrum disorder: Study to Explore Early Development (SEED). Autism Res.

[30] Kohler-Forsberg O (2019). A Nationwide Study in Denmark of the Association Between Treated Infections and the Subsequent Risk of Treated Mental Disorders in Children and Adolescents. JAMA Psychiatry.

[31] Idring S (2012). Autism spectrum disorders in the Stockholm Youth Cohort: design, prevalence and validity. PLoS One.

[32] Ludvigsson JF, Otterblad-Olausson P, Pettersson BU, Ekbom A (2009). The Swedish personal identity number: possibilities and pitfalls in healthcare and medical research. Eur J Epidemiol.

[33] Gardner RM (2020). The Association of Paternal IQ With Autism Spectrum Disorders and Its Comorbidities: A Population-Based Cohort Study. J Am Acad Child Adolesc Psychiatry.

[34] Blomstrom A (2014). Hospital admission with infection during childhood and risk for psychotic illness--a population-based cohort study. Schizophr Bull.

[35] Khandaker GM (2018). Association of Childhood Infection With IQ and Adult Nonaffective Psychosis in Swedish Men: A Population-Based Longitudinal Cohort and Co-relative Study. JAMA Psychiatry.

[36] Mackay E, Dalman C, Karlsson H, Gardner RM (2017). Association of Gestational Weight Gain and Maternal Body Mass Index in Early Pregnancy With Risk for Nonaffective Psychosis in Offspring. JAMA Psychiatry.

[37] Sanchez-Luna M, Medrano C, Lirio J, Group R-S (2017). Down syndrome as risk factor for respiratory syncytial virus hospitalization: A prospective multicenter epidemiological study. Influenza Other Respir Viruses.

[38] Soorya L (2013). Prospective investigation of autism and genotype-phenotype correlations in 22q13 deletion syndrome and SHANK3 deficiency. Mol Autism.

[39] Ivarsson SA, Bjerre I, Vegfors P, Ahlfors K (1990). Autism as one of several disabilities in two children with congenital cytomegalovirus infection. Neuropediatrics..

[40] Chess S, Fernandez P, Korn S (1978). Behavioral consequences of congenital rubella. J Pediatr.

[41] Deykin EY, MacMahon B (1979). Viral exposure and autism. Am J Epidemiol.

[42] Christie D (2017). Impact of meningitis on intelligence and development: A systematic review and meta-analysis. PLoS One.

[43] Engman ML (2008). Neuropsychologic outcomes in children with neonatal herpes encephalitis. Pediatr Neurol.

[44] Pace D, Pollard AJ (2012). Meningococcal disease: clinical presentation and sequelae. Vaccine..

[45] Grimwood K (2000). Twelve year outcomes following bacterial meningitis: further evidence for persisting effects. Arch Dis Child.

[46] Kariuki M (2016). Hospital admission for infection during early childhood influences developmental vulnerabilities at age 5 years. J Paediatr Child Health.

[47] Benros ME (2015). The Association between Infections and General Cognitive Ability in Young Men - A Nationwide Study. PLoS One.

[48] Verstegen RH (2013). Significant impact of recurrent respiratory tract infections in children with Down syndrome. Child Care Health Dev.

[49] Mason-Brothers A (1993). The UCLA-University of Utah epidemiologic survey of autism: Recurrent infections. Eur Child Adolesc Psychiatry.

[50] Badran HS, Abulnasr KM, Abd El Hameed Nasser S (2013). Effect of recurrent otitis media on language profile in children with fragile x syndrome. Clin Med Insights Ear Nose Throat.

[51] Fowler A, Stodberg T, Eriksson M, Wickstrom R (2008). Childhood encephalitis in Sweden: etiology, clinical presentation and outcome. Eur J Paediatr Neurol.

[52] Semple BD (2013). Brain development in rodents and humans: Identifying benchmarks of maturation and vulnerability to injury across species. Prog Neurobiol.

[53] Boucher A (2017). Epidemiology of infectious encephalitis causes in 2016. Med Mal Infect.

[54] Venkatesan A, Jagdish B (2019). Imaging in Encephalitis. Semin Neurol.

[55] Beattie GC (2013). Encephalitis with thalamic and basal ganglia abnormalities: etiologies, neuroimaging, and potential role of respiratory viruses. Clin Infect Dis.

[56] Hanninen P (1980). Involvement of the central nervous system in acute, uncomplicated measles virus infection. J Clin Microbiol.

[57] Thurm A (2019). State of the Field: Differentiating Intellectual Disability From Autism Spectrum Disorder. Front Psychiatry.

[58] Mora S, Martin-Gonzalez E, Flores P, Moreno M (2020). Neuropsychiatric consequences of childhood group A streptococcal infection: A systematic review of preclinical models. Brain Behav Immun.

[59] Warre-Cornish K (2020). Interferon-gamma signaling in human iPSC-derived neurons recapitulates neurodevelopmental disorder phenotypes. Sci Adv.

[60] Gandal MJ (2018). Shared molecular neuropathology across major psychiatric disorders parallels polygenic overlap. Science..

[61] Nazeen S, Palmer NP, Berger B, Kohane IS (2016). Integrative analysis of genetic data sets reveals a shared innate immune component in autism spectrum disorder and its co-morbidities. Genome Biol.

[62] Voineagu I (2011). Transcriptomic analysis of autistic brain reveals convergent molecular pathology. Nature..

[63] Ashwood P, Wills S, Van de Water J (2006). The immune response in autism: a new frontier for autism research. J Leukoc Biol.

[64] Filiano AJ (2016). Unexpected role of interferon-gamma in regulating neuronal connectivity and social behaviour. Nature..

[65] Calder PC (2013). Feeding the immune system. Proc Nutr Soc.

[66] Powanda MC, Beisel WR (2003). Metabolic effects of infection on protein and energy status. J Nutr.

[67] Hwang AE (2013). Childhood infections and adult height in monozygotic twin pairs. Am J Epidemiol.

[68] Cusick SE, Georgieff MK (2016). The Role of Nutrition in Brain Development: The Golden Opportunity of the "First 1000 Days". J Pediatr.

[69] Lee BK (2021). Developmental vitamin D and autism spectrum disorders: findings from the Stockholm Youth Cohort. Mol Psychiatry.

[70] Wiegersma AM (2019). Association of Prenatal Maternal Anemia With Neurodevelopmental Disorders. JAMA Psychiatry.

