# Supplementary material for: Childhood infections and autism spectrum disorders and/or intellectual disability: a register-based cohort study
Source: J Neurodev Disord. 2022 Feb 13;14:12. doi: 10.1186/s11689-022-09422-4 (PMC8903600; doi:10.1186/s11689-022-09422-4)
Supplement: Supplementary file 1 — Additional file 1: Figure S1. Flow chart delineating the derivation of the populations included in the present study. Figure S2. Infections during childhood and later diagnosis of ASD or ID. Crude associations between specialized care for infections and later, non-mutually exclusive, diagnosis of ASD and ID. Associations between exposure and diagnoses at different age interval are also shown. Comparison between unrelated individuals in the general population are shown (A-H) and comparisons between full biological siblings (I-P). Figure S3. An exploration of the influence of the different potentially confounding factors on effect estimates for childhood infections on risk of the mutually non-exclusive diagnoses of ASD (left) and ID (right). We used survival analysis with the extended Cox regression model to examine the relationship between childhood infections and each outcome adjusting for each potential confounder individually and compared these results to crude (top) and fully adjusted models (bottom). The effect estimates (HR) for infections after each adjustment are presented along with bands representing the 95% confidence intervals. Figure S4. Sex-stratified analyses of the association between childhood infections and the later diagnosis of ASD or ID. Fully adjusted associations between specialized care for infections and later, non-mutually exclusive, diagnosis of ASD and ID among males (top row) and females (bottom row). Associations between exposure and diagnoses at different age interval are also shown. Only comparison between unrelated individuals in the general population are shown. Figure S5. Infections during childhood and mutually exclusive diagnoses. Crude associations between infections between birth and age 18 and later diagnosis of ‘ASD without ID’, ‘ASD with ID’ or ‘ID without ASD’. Associations between exposures and diagnoses at different ages are also shown. Comparisons between unrelated individuals in the general population (A-L) and between f [file 11689_2022_9422_MOESM1_ESM.docx]

Supplementary figures, Karlsson, *et al*., *Childhood infections and risk of ASD/ID*


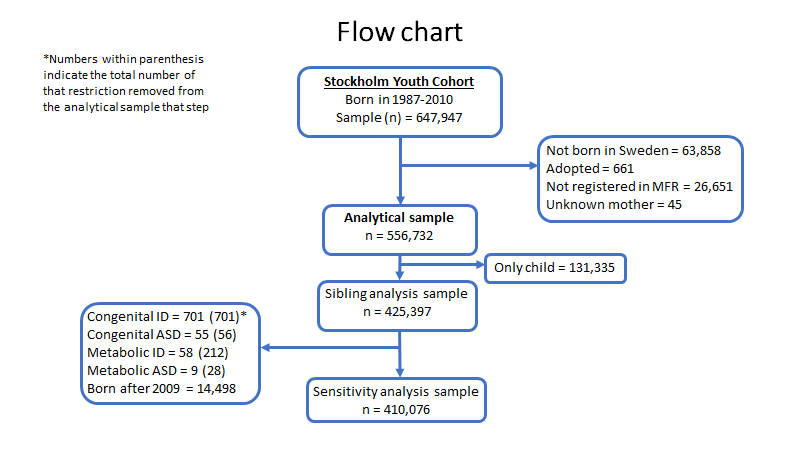
**Figure S1.** Flow chart delineating the derivation of the populations included in the present study.


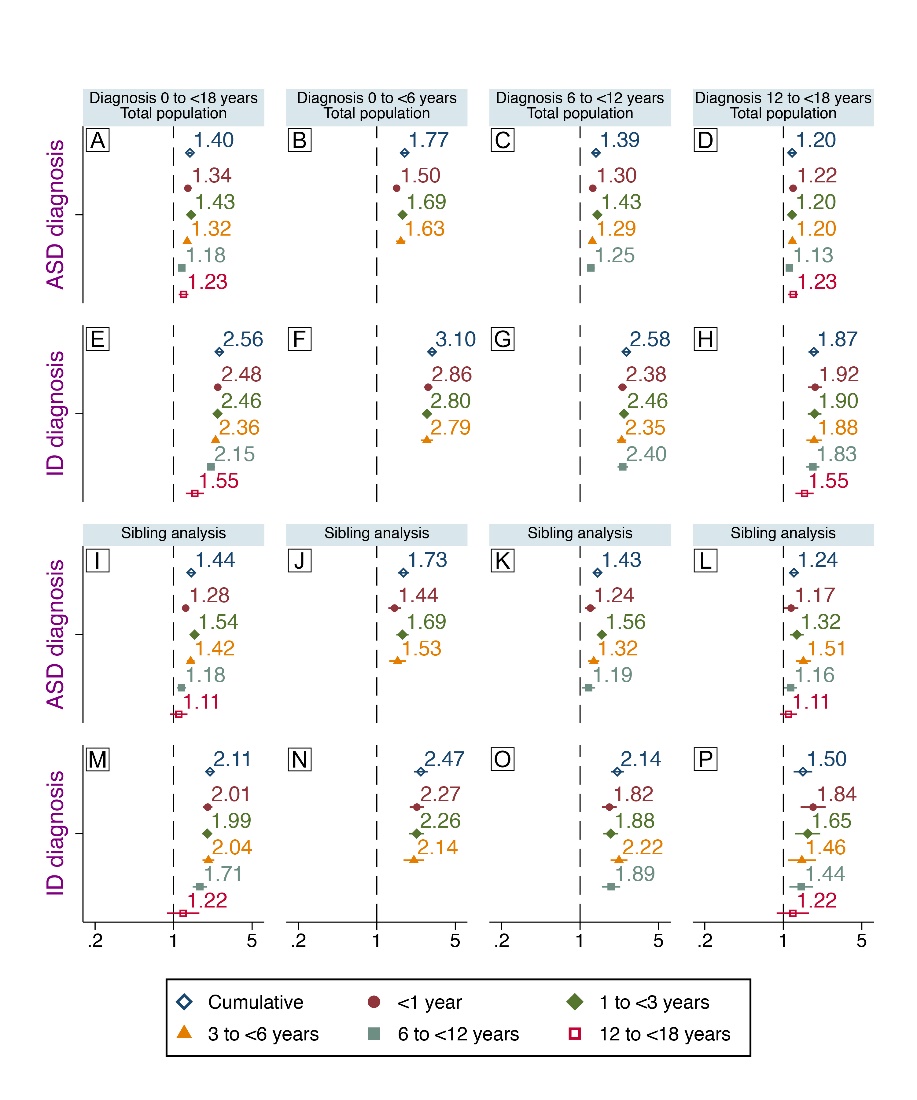

**Figure S2. Infections during childhood and later diagnosis of ASD or ID.** Crude associations between specialized care for infections and later, non-mutually exclusive, diagnosis of ASD and ID. Associations between exposure and diagnoses at different age interval are also shown. Comparison between unrelated individuals in the general population are shown (A-H) and comparisons between full biological siblings (I-P).


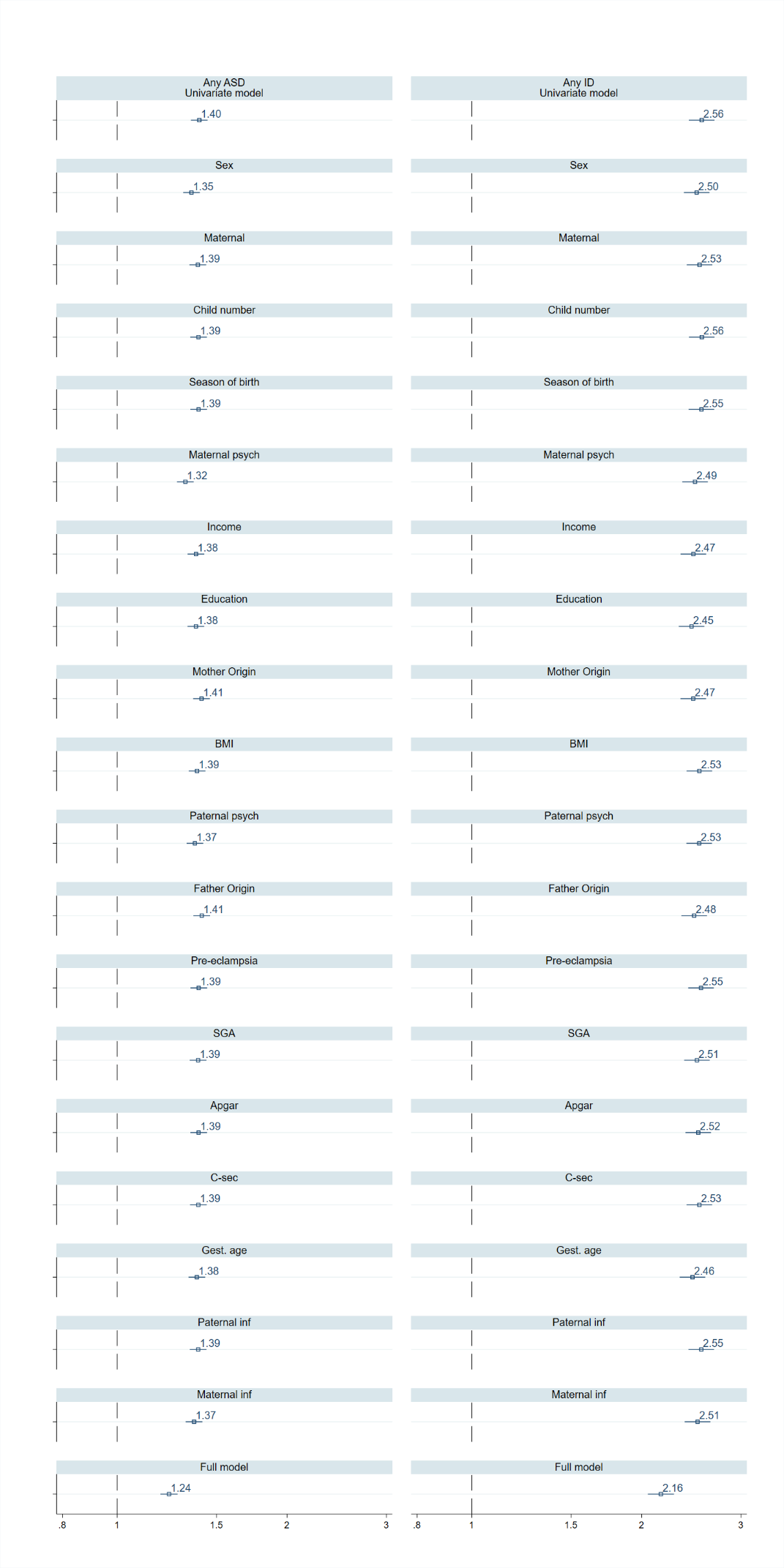


**Figure S3.** An exploration of the influence of the different potentially confounding factors on effect estimates for childhood infections on risk of the mutually non-exclusive diagnoses of ASD (left) and ID (right). We used survival analysis with the extended Cox regression model to examine the relationship between childhood infections and each outcome adjusting for each potential confounder individually and compared these results to crude (top) and fully adjusted models (bottom). The effect estimates (HR) for infections after each adjustment are presented along with bands representing the 95% confidence intervals.


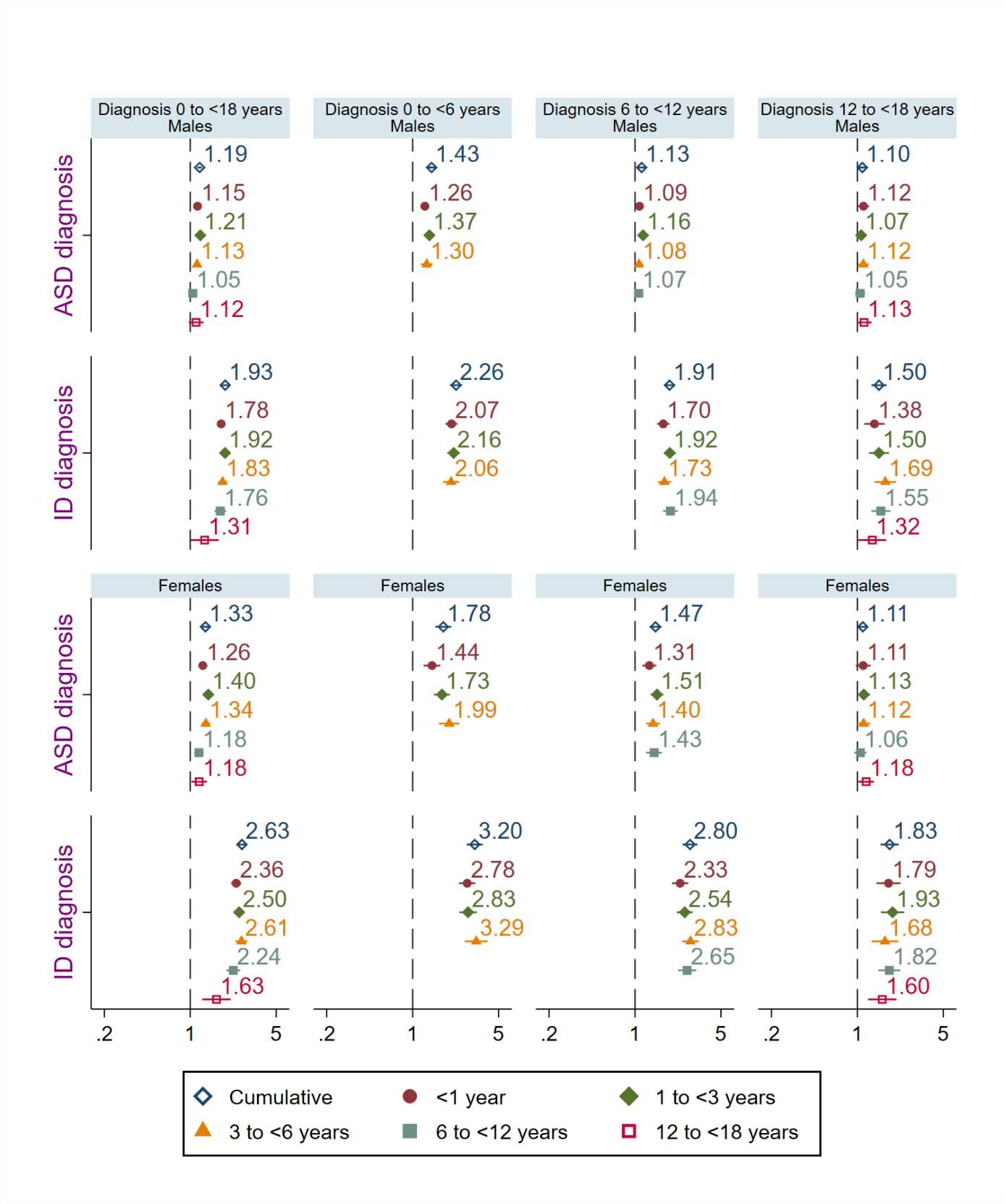


**Figure S4. Sex-stratified analyses of the association between childhood infections and the later diagnosis of ASD or ID.** Fully adjusted associations between specialized care for infections and later, non-mutually exclusive, diagnosis of ASD and ID among males (top row) and females (bottom row). Associations between exposure and diagnoses at different age interval are also shown. Only comparison between unrelated individuals in the general population are shown.


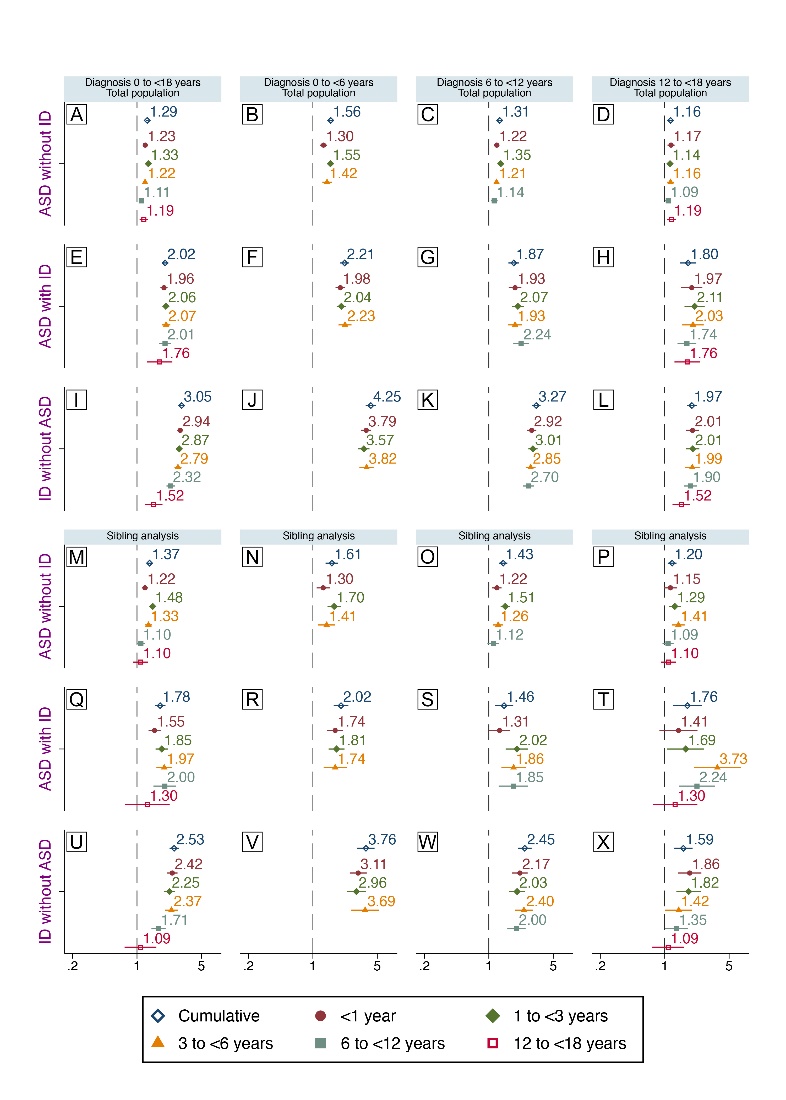


**Figure S5.** **Infections during childhood and mutually exclusive diagnoses.** Crude associations between infections between birth and age 18 and later diagnosis of ‘ASD without ID’, ‘ASD with ID’ or ‘ID without ASD’. Associations between exposures and diagnoses at different ages are also shown. Comparisons between unrelated individuals in the general population (A-L) and between full biological siblings (M-X) are shown.


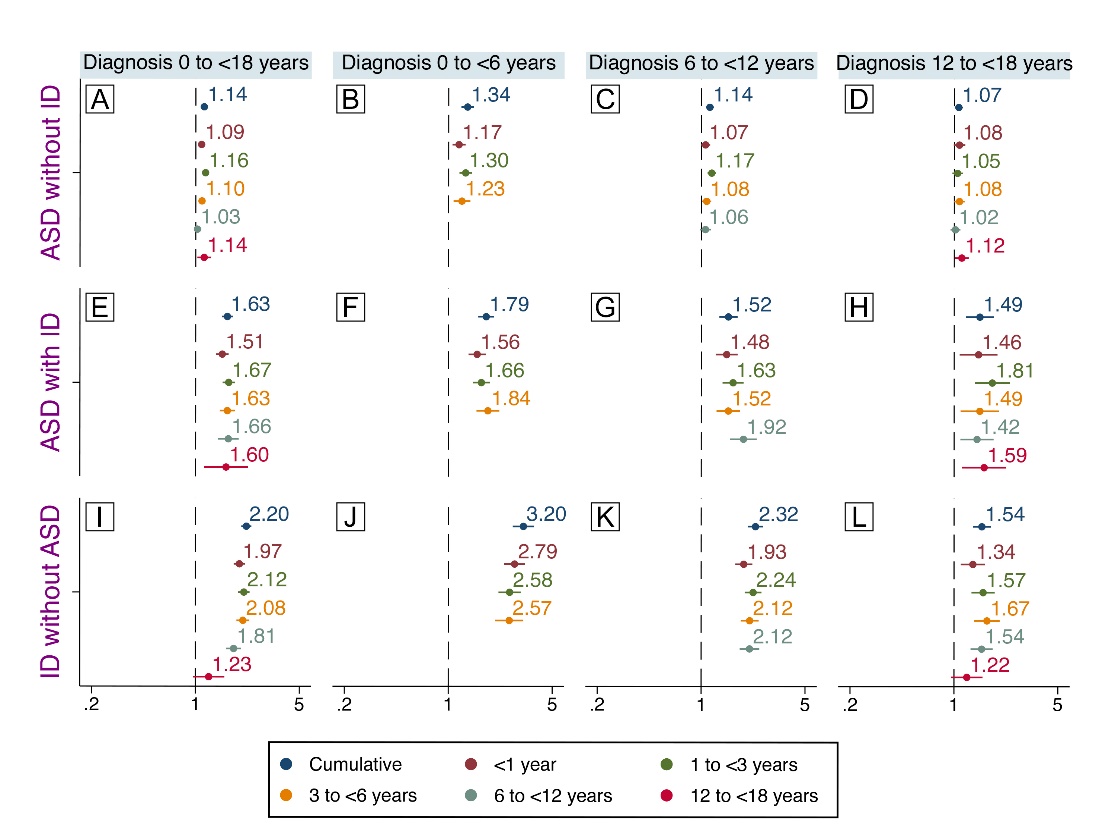


**Figure S6. Infections during childhood and mutually exclusive diagnoses-sensitivity analyses.** Associations between infections between birth and age 18 and later diagnosis of ‘ASD without ID’, ‘ASD with ID’ or ‘ID without ASD’ in the total population following exclusion of individuals with congenital diagnoses and ASD/ID. Associations between exposures and diagnoses at different ages are also shown. Estimates are adjusted for sex, parity, maternal body mass index, pre-eclampsia, parental age, education, income, region of origin, histories of psychiatric illness and infections, season of birth, gestational age at birth, size for gestational age, cesarean section and Apgar score.


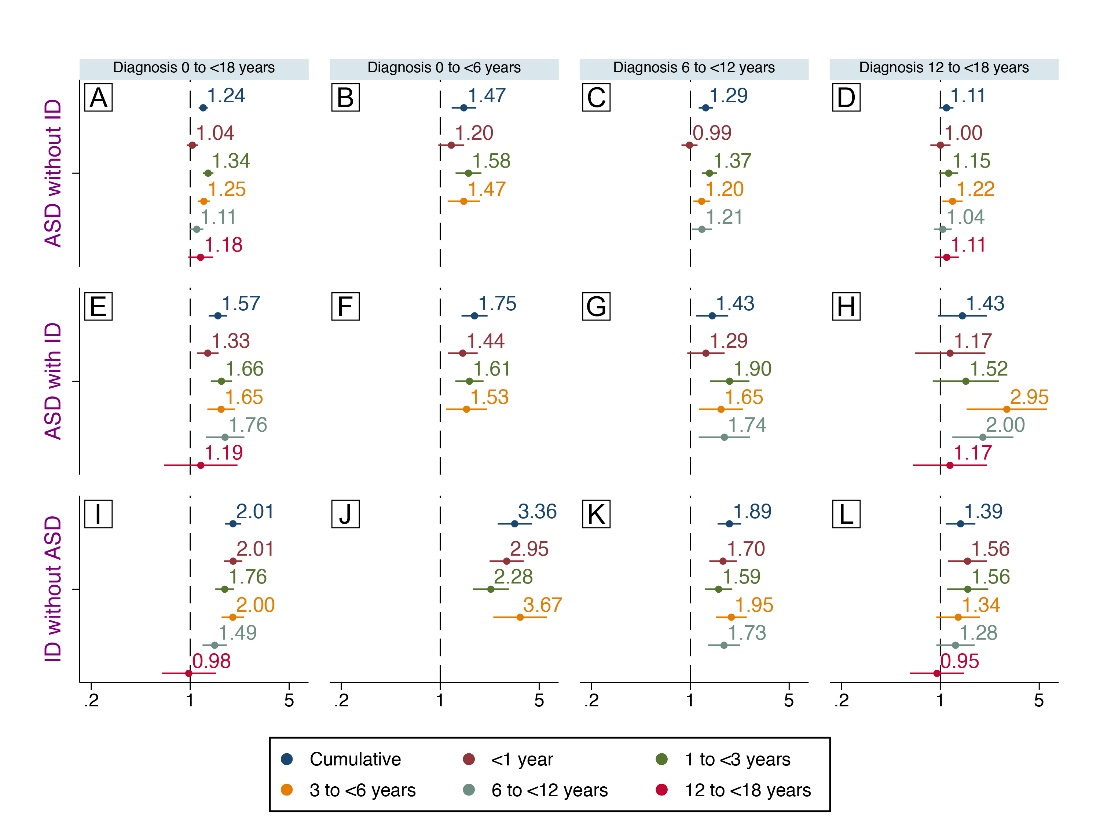


**Figure S7.** **Infections during childhood and mutually exclusive diagnoses-sensitivity analyses.** Associations between infections between birth and age 18 and later diagnosis of ‘ASD without ID’, ‘ASD with ID’ or ‘ID without ASD’ among biological siblings following exclusion of individuals with congenital diagnoses and ASD/ID. Associations between exposures and diagnoses at different ages are also shown. Estimates from are adjusted for sex, parity, gestational age at birth and cesarean section.
