# Supplementary material for: Childhood infections and autism spectrum disorders and/or intellectual disability: a register-based cohort study
Source: J Neurodev Disord. 2022 Feb 13;14:12. doi: 10.1186/s11689-022-09422-4 (PMC8903600; doi:10.1186/s11689-022-09422-4)
Supplement: Supplementary file 2 — Additional file 2: Table S1. ICD codes for infection diagnoses. Table S2. Association between covariates and ASD and by age at first diagnosis. Table S3. Association between covariates and ID and by age at first diagnosis. [file 11689_2022_9422_MOESM2_ESM.docx]

**Table S1**: ICD codes for infection diagnoses.

| Any infection | ICD-8 | 006.00-007.99, 009.00-009.98, 084.00-087.99, 099.96-099.99, 110.00-130.10, 130.99-131.99, 136.09, 320.88-320.99, 360.00, 380.02-381.99, 384.00-384.08, 420.00-420.09, 421.98, 422.97-422.99, 462.01, 462.09, 463.09, 466.99, 483.99-486.09, 503.00-503.09, 540.00-540.02, 540.04-540.99, 572,99, 686.00-686.98, 761,40, 763.10, 763.98, 778,60 + ICD-8 codes in ‘Bacterial infection’ and ‘Viral infection’. |
| --- | --- | --- |
|  | ICD-9 | 006-007X, 008W, 009-D, 084-086X, 099E-X, 110-136X, 321A, 321W, 370E-F, X, 372A-D, 380B, C, 381A, 382X, 420- 422X, 462-463, 466-B, 473-X, 483, 485-486, 490, 491B, 540A, X, 572A, 647C, E, W, X, 680A, 711G-X, 727A, 770A, 771C, E-W + ICD-9 codes in ‘Bacterial infection’ and ‘Viral infection’. |
|  | ICD-10 | A06-07.9, A08.5, A09, A59-59.9, A63, A63.8-64, B35-49, B50 -89, B99, G02.1-02.8, G04, G04.9, G05.2, H10.0, H10.3-10.9, H16.2-16.3, H16.9, H32, H60, H60.3, H65.0-65.1, H66.9, I30.0-30.9, I33.0-33.9, I40.0, J02*, J02.8-02.9*, J03*, J03.8-03.9*, J16, J16.8, J18-18.9, J20, J20.8-21, J21.8-21.9, J22, J32-32.9*, J35.0, J37-37.1*, J40-42, K35, K35.9, K75.0, L30.3, M46.5, M65.1, M71.1, O98.3, O98.6-98.9, P23.8-23.9, P37.1-39.9, Z22.4, Z22.8-22.9 + ICD-10 codes in ‘Bacterial infection’ and ‘Viral infection’. |
| Bacterial | ICD-8 | 000.01-005.99, 008.00-008.30, 010.99-018.98, 020.00-039.98, 073.99, 076.99, 079.30, 080.99-083.99, 088.99-104.98, 320.00-320.80, 322.00-322.03, 361.00-361.09, 362.02, 366.00, 369.00, 380.00-380.01, 382.00-383.99, 390.97-392.99, 421.00, 461.00-461.09, 462.02, 463.01, 481,99-482.98, 501.99, 508.00-508.02, 510.01-510.09, 511.10, 513.99, 522.50, 527.30, 528.00, 528.30, 540.03, 562.00-562.19, 566.00-566.01, 567.00-567.02, 569.00, 577.01, 590.00-590.99, 595.00-595.02, 597.00, 599.02, 611.00, 611.01, 612.01-614.99, 616.00-616.03, 620.00-620.99, 622.00-622.19, 629.40, 630.00-630.09, 635.00-636.09, 645.90-645.91, 670.00-670.09, 678.02, 680.00-682.99, 684.00-684.09, 710.00-710.09, 720.00-720.29, 732.99, 761,00, 763.00, 998.50, 999.30 |
|  | ICD-9 | 001-005X, 008A-F, 010-041X, 073, 076, 078D, J, 790H, 080-083X, 087-099D, 100-104, 245A, 254B, 320-X, 324-X, 360A, 373B, 375D, 376A, 382A-E, 383A-X, 390-392X, 421A, 461-X, 475, 481-482X, 510-X, 511B, 513-B, 522E, H, 526E, 527D, 528A, D, 540B, 562-B, 566, 567-C, 569F, 575A, 590-X, 597A, 595-D, X, 597W, 599A, 611A, 614-F, W-X, 615A, X, 616-X, 634A, 635A, 636A, 637A, 638A, 639A, 646F, G, 647A, B, D, 658E, 659D, 670, 675-B, W-X, 681-686X, 711A, E, 728A, 729E, 730-D, X, 771D, 996G, 998F, 999D |
|  | ICD-10 | A00-05.9, A15-17.9, A20-28.9, A30 -58, A65 -79.9, B95-96.8, E06.0, E32.1, G00-00.9, G01, G04.2, G05.0, G06-06.2*, G07, H00.0, H01.0, H04.3, H05.0, H44.0, H60.0-60.1, H66.0-66.4, H70.0-70.9, I00-02.9, J01-01.9*, J02.0, J03.0, J13-15.9, J16.0, J20.0-20.2, J34.0, J36*, J39.0-39.1, J85.1-85.3, J86-86.9*, K04.6-04.7, K05.2, K11.3, K12.2, K14.0, K35.1, K57-57.9, K61-61.4, K63.0, K65.0*, K81.0, K85, L00 -08.9, M00-00.9, M46.3*, M60.0*, M86-86.9*, N10-12*, N13.6*, N15.1, N15.9, N30-30.3*, N30.8-30.9*, N34-34.1*, N39.0*, , N61, N70-76.8*, N98.0, O07.0, O07.5, O08.0, O23-23.9, O41.1, O75.3, O85-86.8*, O91-91.1, O98.0-98.2, P23.1-23.6, P36, P37.0, T80.2, T81.4, T82.6-82.7, T83.5-83.6, T84.5-84.7, T85.7, T88.0, Z22.0-22.3 |
| Viral | ICD-8 | 008.80-008.98, 040.00-043.99, 045.00-065.99, 067.00-072.09, 074.00-075.09, 078.00-079.20, 079.40-079.99, 099.92, 460.99, 464.01-480,99, 508.03, 761.20, 761.30 |
|  | ICD-9 | 008H-M, 045-066, 070-072X, 074-075, 077-078H, 078W-079X, 279K, 321B-H, 323A, 323C-D, 460, 464-465X, 480-X, 487-W, 647F, G, 711F, 771A, B, 790W |
|  | ICD-10 | A08-08.4, A60-60.9, A63.0, A80-89, A90-99, B00-06.0, B06.8-09, B15-19.9, B20-24, B25-34, B97-97.8, G02.0, G05.1, J00, J04-06.9*, J10-11.8, J12-12.9, J20.3-20.7, J21.0, O35.3, O98.4-98.5, P23.0, P35, Z21, Z22.5-22.6 |
| CNS | ICD-8 | 013.00-013.99, 027.01, 036.00, 090.40, 094.00-094.98, 320.00-320.80, 322.00-322.03, 392.99, 040.00-043.99, 045.00-046.99, 052.00, 054.04, 062.00-065.99, 071.99, 072.01, 075.02, 079.20, 474.99, 084.00, 320.88-320.99 |
|  | ICD-9 | 013-X, 036A, B, 090E, 094-X, 320-X, 324-X, 392-X, 045-049X, 054D, 052B, 053A, 055A, 056A, 071, 072B, C, 321B-H, 323A, 323C, D, 006F, 321A, 321W |
|  | ICD-10 | A02.2 (if G01), A17-17.9, A20.3, A22.8, A32.1, A39.0, A39.8 (if G05), A50.4 (if G05.0 or G01), A51.4 (if G01), A52.1 (if G05.0, G01 or F02.8), A54.8 (if G07 or G01), A69.2 (if G01), G00-00.9, G01, G04.2, G05.0, G06-06.2, G07, I02-02.9, A80-89, B00.3-00.4, B01.0-01.1, B02.0-02.1, B05.0-05.1, B06.0, B26.1-26.2, G02.0, G05.1, B58.2, A06.6, B37.5, B38.4, B43.1, B45.1, B46.1, B50.0, B57.4, B60.2, B69.0, B83.2, G02.1-02.8, G04, G04.9, G05.2 |
| Respiratory | ICD-8 | 010-012, 020.10, 461.00-461.09, 462.02, 463.01, 481,99-482.98, 501.99, 508.00-508.02, 510.01-510.09, 511.10, 513.99, 460.99, 464.01-464.09, 465.99, 470.99-473.99, 480,99, 508.03, 462.01, 462.09, 463.09, 466.99, 483.99-486.09, 502.00-503.09, 519.92, 490.99-491.09 |
|  | ICD-9 | 010-012W, 031A, 033-034B, 052A, 055B, 112E, 122B, 460-466, 475, 481-482X, 510-X, 511B, 513-B, 480-X, 487-W, 462, 463, 466-B, 473-X, 483, 485, 486, 490, 491B |
|  | ICD-10 | A15-16, A20.2, A21.2, A22.1, A31.0, A37, A38, A48.1, B00.2, B01.2, B05.2, B27, B37.1, B39-42, B44, B45.0, B46.0, B58.3, B59, J01-01.9, J02.0, J03.0, J13-15.9, J16.0, J20.0-20.2, J34.0, J36, J39.0-39.1, J85.1-85.3, J86-86.9, J00, J04-06.9, J10-11.8, J12-12.9, J20.3-20.7, J21.0, , J02, J02.8-02.9, J03, J03.8-03.9, J16, J16.8, J18-18.9, J20, J20.8-21, J21.8-21.9, J22, J32-32.9, J35.0, J37-37.1, J40-42 |
| Skin | ICD-8 | 017.01-017.09, 110-111, 050-057, 680.00-680.90, 681.00-682.99, 684.00-684.09, 686.00-686.98 |
|  | ICD-9 | 017A, 031B, 050-057, 074D, 091D, 110-111, 112D, 681-682X, 683, 684, 685-686X, 680A |
|  | ICD-10 | A18.4, A20.0, A22.0, A26.0, A31.1, A32, A36.3, B00-09, B35-36, B37.2, B43.0, B43.2, B45.2, B46.3, B55.1, L00, L01-01.1, L02-02.9, L03-03.9, L04-08.9, L70.0, L30.3 |
| Genitourinary | ICD-8 | 090-099, 016, 054.02, 590.00-590.99, 595.00-595.02, 597.00, 599.02, 601.00, 604.00, 604.01, 607.30, 611.00, 611.01, 612.01-614.99, 616.00-616.03, 620.00-620.99, 622.00-622.19, 629.40 |
|  | ICD-9 | 016, 054B, 112B, C, 090-099, 131A, 590-X, 597A, 595-D, X, 597W, 599A, 601-D, 603B, 604A, 604X, 607B, C, 608A, E, 611A, 614-F, W-X, 615A, X, 616-X |
|  | ICD-10 | A18.0-18.1, A50-64, A70-74, B37.3-37.4, N10-12, N13.6, N15.1, N15.9, N30-30.3, N30.8-30.9, N34-34.1, N39.0, N41-41.3, N43.1, N45.0-45.9, N48.1-48.2, N49-49.9, N61, N70-76.8, N98.0 |
| Gastrointestinal | ICD-8 | 000-009, 014, 039.92, 127.99, 522.50, 527.30, 528.00, 528.30, 562.00-562.19, 566.00-566.01, 567.00-567.02, 569.00, 577.01, 540.00-540.99, 572,99 |
|  | ICD-9 | 001-009, 123, 123, 127, 129, 014, 522E, H, 526E, 527D, 528A, D, 540B, 562-B, 566, 567-C, 569F, 575A, 540A, X, 572A |
|  | ICD-10 | 001-009, 123, 123, 127, 129, 014, 522E, H, 526E, 527D, 528A, D, 540B, 562-B, 566, 567-C, 569F, 575A, 540A, X, 572A |

¹ The Swedish ICD coding systems were used the following years: ICD-8 1969-86, ICD-9 1987-96 and ICD-10 1997-.

* The additional codes, B95-97.8, determines the infecting organism

**Table S2. Association between covariates and ASD and by age at first diagnosis.**

|  |  | ***No ASD*** | ***ASD*** | ***p-value*** | ***0-<6 years*** | ***6-<12 years*** | ***12-<18 years*** | ***p-value*** |
| --- | --- | --- | --- | --- | --- | --- | --- | --- |
| **N** |  | 542127 | 14605 |  | 3704 | 5845 | 5056 |  |
| **Sex** | Male | 275164 (50.8%) | 10290 (70.5%) | <0.001 | 2870 (77.5%) | 4446 (76.1%) | 2974 (58.8%) | <0.001 |
|  | Female | 266963 (49.2%) | 4315 (29.5%) |  | 834 (22.5%) | 1399 (23.9%) | 2082 (41.2%) |  |
| **Birth order** | First | 244369 (45.1%) | 7051 (48.3%) | <0.001 | 1868 (50.4%) | 2876 (49.2%) | 2307 (45.6%) | <0.001 |
|  | Second | 196328 (36.2%) | 4832 (33.1%) |  | 1196 (32.3%) | 1906 (32.6%) | 1730 (34.2%) |  |
|  | ≥Third | 101430 (18.7%) | 2722 (18.6%) |  | 640 (17.3%) | 1063 (18.2%) | 1019 (20.2%) |  |
| **Birth season** | Dec-Feb | 127267 (23.5%) | 3370 (23.1%) | <0.001 | 821 (22.2%) | 1402 (24.0%) | 1147 (22.7%) | <0.001 |
|  | Mar-May | 146735 (27.1%) | 3690 (25.3%) |  | 912 (24.6%) | 1423 (24.3%) | 1355 (26.8%) |  |
|  | Jun-Aug | 140652 (25.9%) | 3809 (26.1%) |  | 996 (26.9%) | 1483 (25.4%) | 1330 (26.3%) |  |
|  | Sep-Nov | 127473 (23.5%) | 3736 (25.6%) |  | 975 (26.3%) | 1537 (26.3%) | 1224 (24.2%) |  |
| **GA** | Preterm | 30044 (5.5%) | 1141 (7.8%) | <0.001 | 343 (9.3%) | 459 (7.9%) | 339 (6.7%) | <0.001 |
|  | Term | 507893 (93.7%) | 13338 (91.3%) |  | 3339 (90.1%) | 5343 (91.4%) | 4656 (92.1%) |  |
|  | Post term | 3279 (0.6%) | 96 (0.7%) |  | 20 (0.5%) | 32 (0.5%) | 44 (0.9%) |  |
|  | Missing | 911 (0.2%) | 30 (0.2%) |  | 2 (0.1%) | 11 (0.2%) | 17 (0.3%) |  |
| **Size for GA** | Small | 12569 (2.3%) | 540 (3.7%) | <0.001 | 177 (4.8%) | 203 (3.5%) | 160 (3.2%) | <0.001 |
|  | Normal | 495982 (91.5%) | 12942 (88.6%) |  | 3237 (87.4%) | 5186 (88.7%) | 4519 (89.4%) |  |
|  | Large | 16030 (3.0%) | 592 (4.1%) |  | 153 (4.1%) | 240 (4.1%) | 199 (3.9%) |  |
|  | Missing | 17546 (3.2%) | 531 (3.6%) |  | 137 (3.7%) | 216 (3.7%) | 178 (3.5%) |  |
| **Cesarean delivery** | No | 488835 (90.2%) | 12764 (87.4%) | <0.001 | 3126 (84.4%) | 5091 (87.1%) | 4547 (89.9%) | <0.001 |
|  | Yes | 35746 (6.6%) | 1310 (9.0%) |  | 441 (11.9%) | 538 (9.2%) | 331 (6.5%) |  |
|  | Missing | 17546 (3.2%) | 531 (3.6%) |  | 137 (3.7%) | 216 (3.7%) | 178 (3.5%) |  |
| **Low Apgar score (<7)** | No | 530639 (97.9%) | 14194 (97.2%) | <0.001 | 3585 (96.8%) | 5693 (97.4%) | 4916 (97.2%) | <0.001 |
|  | Yes | 4856 (0.9%) | 223 (1.5%) |  | 85 (2.3%) | 78 (1.3%) | 60 (1.2%) |  |
|  | Missing | 6632 (1.2%) | 188 (1.3%) |  | 34 (0.9%) | 74 (1.3%) | 80 (1.6%) |  |
| **Pre-eclampsia** | No | 527700 (97.3%) | 14057 (96.2%) | <0.001 | 3542 (95.6%) | 5633 (96.4%) | 4882 (96.6%) | <0.001 |
|  | Yes | 14427 (2.7%) | 548 (3.8%) |  | 162 (4.4%) | 212 (3.6%) | 174 (3.4%) |  |
| **Maternal BMI** | Underweight | 14729 (2.7%) | 371 (2.5%) | <0.001 | 100 (2.7%) | 144 (2.5%) | 127 (2.5%) | <0.001 |
|  | Normal weight | 274989 (50.7%) | 6885 (47.1%) |  | 1900 (51.3%) | 2672 (45.7%) | 2313 (45.7%) |  |
|  | Overweight | 78155 (14.4%) | 2600 (17.8%) |  | 705 (19.0%) | 1063 (18.2%) | 832 (16.5%) |  |
|  | Obese | 26353 (4.9%) | 1217 (8.3%) |  | 338 (9.1%) | 531 (9.1%) | 348 (6.9%) |  |
|  | Missing | 147901 (27.3%) | 3532 (24.2%) |  | 661 (17.8%) | 1435 (24.6%) | 1436 (28.4%) |  |
| **Maternal age, years** | <25 | 80391 (14.8%) | 2301 (15.8%) | <0.001 | 504 (13.6%) | 903 (15.4%) | 894 (17.7%) | <0.001 |
|  | 25-29 | 159722 (29.5%) | 4199 (28.8%) |  | 994 (26.8%) | 1695 (29.0%) | 1510 (29.9%) |  |
|  | 30-34 | 188569 (34.8%) | 4781 (32.7%) |  | 1211 (32.7%) | 1955 (33.4%) | 1615 (31.9%) |  |
|  | 35-39 | 93935 (17.3%) | 2662 (18.2%) |  | 783 (21.1%) | 1040 (17.8%) | 839 (16.6%) |  |
|  | >39 | 19510 (3.6%) | 662 (4.5%) |  | 212 (5.7%) | 252 (4.3%) | 198 (3.9%) |  |
| **Paternal age, years** | <25 | 36915 (6.9%) | 1147 (7.9%) | <0.001 | 205 (5.6%) | 462 (8.0%) | 480 (9.6%) | <0.001 |
|  | 25-29 | 117557 (21.9%) | 3112 (21.6%) |  | 676 (18.6%) | 1263 (21.8%) | 1173 (23.4%) |  |
|  | 30-34 | 180109 (33.6%) | 4601 (31.9%) |  | 1127 (31.0%) | 1904 (32.9%) | 1570 (31.4%) |  |
|  | 35-39 | 124697 (23.2%) | 3227 (22.4%) |  | 904 (24.9%) | 1275 (22.0%) | 1048 (20.9%) |  |
|  | >39 | 77233 (14.4%) | 2343 (16.2%) |  | 718 (19.8%) | 891 (15.4%) | 734 (14.7%) |  |
| **Mother's region of origin** | Sweden | 397154 (73.3%) | 10919 (74.8%) | <0.001 | 2359 (63.7%) | 4546 (77.8%) | 4014 (79.4%) | <0.001 |
|  | Nordics | 14984 (2.8%) | 451 (3.1%) |  | 58 (1.6%) | 186 (3.2%) | 207 (4.1%) |  |
|  | West Eu and NA | 23954 (4.4%) | 552 (3.8%) |  | 178 (4.8%) | 215 (3.7%) | 159 (3.1%) |  |
|  | Africa | 24973 (4.6%) | 642 (4.4%) |  | 332 (9.0%) | 181 (3.1%) | 129 (2.6%) |  |
|  | Asia and Oceania | 11384 (2.1%) | 278 (1.9%) |  | 146 (3.9%) | 77 (1.3%) | 55 (1.1%) |  |
|  | Middle-East | 45453 (8.4%) | 969 (6.6%) |  | 380 (10.3%) | 352 (6.0%) | 237 (4.7%) |  |
|  | East Eu and Russia | 7262 (1.3%) | 200 (1.4%) |  | 65 (1.8%) | 75 (1.3%) | 60 (1.2%) |  |
|  | South America | 11315 (2.1%) | 417 (2.9%) |  | 112 (3.0%) | 161 (2.8%) | 144 (2.8%) |  |
|  | Unknown/Missing | 5648 (1.0%) | 177 (1.2%) |  | 74 (2.0%) | 52 (0.9%) | 51 (1.0%) |  |
| **Father's region of origin** | Sweden | 406563 (75.0%) | 11253 (77.0%) | <0.001 | 2346 (63.3%) | 4729 (80.9%) | 4178 (82.6%) | <0.001 |
|  | Nordics | 19321 (3.6%) | 495 (3.4%) |  | 84 (2.3%) | 179 (3.1%) | 232 (4.6%) |  |
|  | West Eu and NA | 15974 (2.9%) | 366 (2.5%) |  | 124 (3.3%) | 140 (2.4%) | 102 (2.0%) |  |
|  | Africa | 21694 (4.0%) | 525 (3.6%) |  | 323 (8.7%) | 136 (2.3%) | 66 (1.3%) |  |
|  | Asia and Oceania | 17133 (3.2%) | 427 (2.9%) |  | 206 (5.6%) | 113 (1.9%) | 108 (2.1%) |  |
|  | Middle-East | 39176 (7.2%) | 787 (5.4%) |  | 338 (9.1%) | 289 (4.9%) | 160 (3.2%) |  |
|  | East Eu and Russia | 11764 (2.2%) | 364 (2.5%) |  | 146 (3.9%) | 137 (2.3%) | 81 (1.6%) |  |
|  | South America | 10485 (1.9%) | 388 (2.7%) |  | 137 (3.7%) | 122 (2.1%) | 129 (2.6%) |  |
|  | Unknown/Missing | 17 (<1%) | 0 (0.0%) |  | 0 (0.0%) | 0 (0.0%) | 0 (0.0%) |  |
| **Maternal psychiatric diagnosis** | No | 309103 (57.0%) | 5492 (37.6%) | <0.001 | 1643 (44.4%) | 2067 (35.4%) | 1782 (35.2%) | <0.001 |
|  | Yes | 233024 (43.0%) | 9113 (62.4%) |  | 2061 (55.6%) | 3778 (64.6%) | 3274 (64.8%) |  |
| **Paternal psychiatric diagnosis** | No | 395044 (72.9%) | 8982 (61.5%) | <0.001 | 2405 (64.9%) | 3557 (60.9%) | 3020 (59.7%) | <0.001 |
|  | Yes | 147083 (27.1%) | 5623 (38.5%) |  | 1299 (35.1%) | 2288 (39.1%) | 2036 (40.3%) |  |
| **Maternal infection** | No | 216953 (40.0%) | 4724 (32.3%) | <0.001 | 1052 (28.4%) | 1806 (30.9%) | 1866 (36.9%) | <0.001 |
|  | Yes | 325174 (60.0%) | 9881 (67.7%) |  | 2652 (71.6%) | 4039 (69.1%) | 3190 (63.1%) |  |
| **Paternal infection** | No | 313856 (57.9%) | 7824 (53.6%) | <0.001 | 1899 (51.3%) | 3078 (52.7%) | 2847 (56.3%) | <0.001 |
|  | Yes | 228271 (42.1%) | 6781 (46.4%) |  | 1805 (48.7%) | 2767 (47.3%) | 2209 (43.7%) |  |
| **Family income** | Low (0<20%) | 77873 (14.4%) | 2024 (13.9%) | <0.001 | 646 (17.4%) | 731 (12.5%) | 647 (12.8%) | <0.001 |
|  | 20<40% | 113692 (21.0%) | 3720 (25.5%) |  | 933 (25.2%) | 1503 (25.7%) | 1284 (25.4%) |  |
|  | 40<60% | 117331 (21.6%) | 3484 (23.9%) |  | 825 (22.3%) | 1375 (23.5%) | 1284 (25.4%) |  |
|  | 60<80% | 116754 (21.5%) | 2966 (20.3%) |  | 674 (18.2%) | 1247 (21.3%) | 1045 (20.7%) |  |
|  | High (80-100%) | 115761 (21.4%) | 2392 (16.4%) |  | 618 (16.7%) | 982 (16.8%) | 792 (15.7%) |  |
|  | Missing | 716 (0.1%) | 19 (0.1%) |  | 8 (0.2%) | 7 (0.1%) | 4 (0.1%) |  |
| **Parental education** | <10 years | 33479 (6.2%) | 1014 (6.9%) | <0.001 | 234 (6.3%) | 369 (6.3%) | 411 (8.1%) | <0.001 |
|  | 10-12 | 206592 (38.1%) | 6208 (42.5%) |  | 1292 (34.9%) | 2537 (43.4%) | 2379 (47.1%) |  |
|  | >12 | 300631 (55.5%) | 7357 (50.4%) |  | 2165 (58.5%) | 2931 (50.1%) | 2261 (44.7%) |  |
|  | Missing | 1425 (0.3%) | 26 (0.2%) |  | 13 (0.4%) | 8 (0.1%) | 5 (0.1%) |  |

Autism spectrum disorder (ASD), body mass index (BMI), Gestational age (GA), North America (NA), Europe (Eu)

**Table S3. Association between covariates and ID and by age at first diagnosis.**

|  |  | ***No ID*** | ***ID*** | ***p-value*** | ***0-<6 years*** | ***6-<12 years*** | ***12-<18 years*** | ***p-value*** |
| --- | --- | --- | --- | --- | --- | --- | --- | --- |
| **N** |  | 550216 | 6516 |  | 2410 | 2710 | 1396 |  |
| **Sex** | Male | 281322 (51.1%) | 4132 (63.4%) | <0.001 | 1556 (64.6%) | 1754 (64.7%) | 822 (58.9%) | <0.001 |
|  | Female | 268894 (48.9%) | 2384 (36.6%) |  | 854 (35.4%) | 956 (35.3%) | 574 (41.1%) |  |
| **Birth order** | First born | 248771 (45.2%) | 2649 (40.7%) | <0.001 | 1003 (41.6%) | 1103 (40.7%) | 543 (38.9%) | <0.001 |
|  | Second born | 198901 (36.1%) | 2259 (34.7%) |  | 843 (35.0%) | 932 (34.4%) | 484 (34.7%) |  |
|  | Third born or later | 102544 (18.6%) | 1608 (24.7%) |  | 564 (23.4%) | 675 (24.9%) | 369 (26.4%) |  |
| **Birth season** | Dec-Feb | 129074 (23.5%) | 1563 (24.0%) | 0.21 | 569 (23.6%) | 664 (24.5%) | 330 (23.6%) | 0.014 |
|  | Mar-May | 148692 (27.0%) | 1733 (26.6%) |  | 597 (24.8%) | 724 (26.7%) | 412 (29.5%) |  |
|  | Jun-Aug | 142825 (26.0%) | 1636 (25.1%) |  | 654 (27.1%) | 670 (24.7%) | 312 (22.3%) |  |
|  | Sep-Nov | 129625 (23.6%) | 1584 (24.3%) |  | 590 (24.5%) | 652 (24.1%) | 342 (24.5%) |  |
| **GA at birth** | Preterm | 30345 (5.5%) | 840 (12.9%) | <0.001 | 343 (14.2%) | 344 (12.7%) | 153 (11.0%) | <0.001 |
|  | Term | 515635 (93.7%) | 5596 (85.9%) |  | 2041 (84.7%) | 2331 (86.0%) | 1224 (87.7%) |  |
|  | Post term | 3321 (0.6%) | 54 (0.8%) |  | 19 (0.8%) | 21 (0.8%) | 14 (1.0%) |  |
|  | Missing | 915 (0.2%) | 26 (0.4%) |  | 7 (0.3%) | 14 (0.5%) | 5 (0.4%) |  |
| **Size for GA** | Small | 12603 (2.3%) | 506 (7.8%) | <0.001 | 208 (8.6%) | 210 (7.7%) | 88 (6.3%) | <0.001 |
|  | Normal | 503478 (91.5%) | 5446 (83.6%) |  | 1981 (82.2%) | 2270 (83.8%) | 1195 (85.6%) |  |
|  | Large | 16393 (3.0%) | 229 (3.5%) |  | 85 (3.5%) | 88 (3.2%) | 56 (4.0%) |  |
|  | Missing | 17742 (3.2%) | 335 (5.1%) |  | 136 (5.6%) | 142 (5.2%) | 57 (4.1%) |  |
| **Cesarean delivery** | No | 496004 (90.1%) | 5595 (85.9%) | <0.001 | 1991 (82.6%) | 2327 (85.9%) | 1277 (91.5%) | <0.001 |
|  | Yes | 36470 (6.6%) | 586 (9.0%) |  | 283 (11.7%) | 241 (8.9%) | 62 (4.4%) |  |
|  | Missing | 17742 (3.2%) | 335 (5.1%) |  | 136 (5.6%) | 142 (5.2%) | 57 (4.1%) |  |
| **Low Apgar score (<7)** | No | 538709 (97.9%) | 6124 (94.0%) | <0.001 | 2249 (93.3%) | 2558 (94.4%) | 1317 (94.3%) | <0.001 |
|  | Yes | 4824 (0.9%) | 255 (3.9%) |  | 118 (4.9%) | 92 (3.4%) | 45 (3.2%) |  |
|  | Missing | 6683 (1.2%) | 137 (2.1%) |  | 43 (1.8%) | 60 (2.2%) | 34 (2.4%) |  |
| **Pre-eclampsia** | No | 535497 (97.3%) | 6260 (96.1%) | <0.001 | 2306 (95.7%) | 2599 (95.9%) | 1355 (97.1%) | <0.001 |
|  | Yes | 14719 (2.7%) | 256 (3.9%) |  | 104 (4.3%) | 111 (4.1%) | 41 (2.9%) |  |
| **Maternal BMI** | Underweight | 14911 (2.7%) | 189 (2.9%) | <0.001 | 63 (2.6%) | 77 (2.8%) | 49 (3.5%) | <0.001 |
|  | Normal weight | 279127 (50.7%) | 2747 (42.2%) |  | 1163 (48.3%) | 1103 (40.7%) | 481 (34.5%) |  |
|  | Overweight | 79730 (14.5%) | 1025 (15.7%) |  | 447 (18.5%) | 391 (14.4%) | 187 (13.4%) |  |
|  | Obese | 27037 (4.9%) | 533 (8.2%) |  | 211 (8.8%) | 230 (8.5%) | 92 (6.6%) |  |
|  | Missing | 149411 (27.2%) | 2022 (31.0%) |  | 526 (21.8%) | 909 (33.5%) | 587 (42.0%) |  |
| **Maternal age, years** | <25 | 81411 (14.8%) | 1281 (19.7%) | <0.001 | 363 (15.1%) | 534 (19.7%) | 384 (27.5%) | <0.001 |
|  | 25-29 | 161994 (29.4%) | 1927 (29.6%) |  | 663 (27.5%) | 833 (30.7%) | 431 (30.9%) |  |
|  | 30-34 | 191406 (34.8%) | 1944 (29.8%) |  | 780 (32.4%) | 816 (30.1%) | 348 (24.9%) |  |
|  | 35-39 | 95542 (17.4%) | 1055 (16.2%) |  | 457 (19.0%) | 414 (15.3%) | 184 (13.2%) |  |
|  | >39 | 19863 (3.6%) | 309 (4.7%) |  | 147 (6.1%) | 113 (4.2%) | 49 (3.5%) |  |
| **Paternal age, years** | <25 | 37471 (6.9%) | 591 (9.2%) | <0.001 | 137 (5.8%) | 254 (9.5%) | 200 (14.4%) | <0.001 |
|  | 25-29 | 119222 (21.9%) | 1447 (22.5%) |  | 469 (19.8%) | 615 (23.0%) | 363 (26.2%) |  |
|  | 30-34 | 182782 (33.6%) | 1928 (30.0%) |  | 715 (30.2%) | 825 (30.8%) | 388 (28.0%) |  |
|  | 35-39 | 126569 (23.2%) | 1355 (21.1%) |  | 561 (23.7%) | 555 (20.7%) | 239 (17.2%) |  |
|  | >39 | 78467 (14.4%) | 1109 (17.2%) |  | 487 (20.6%) | 426 (15.9%) | 196 (14.1%) |  |
| **Mother's region of origin** | Sweden | 404103 (73.4%) | 3970 (60.9%) | <0.001 | 1362 (56.5%) | 1687 (62.3%) | 921 (66.0%) | <0.001 |
|  | Nordics | 15221 (2.8%) | 214 (3.3%) |  | 43 (1.8%) | 98 (3.6%) | 73 (5.2%) |  |
|  | West Eu and NA | 24207 (4.4%) | 299 (4.6%) |  | 136 (5.6%) | 103 (3.8%) | 60 (4.3%) |  |
|  | Africa | 25044 (4.6%) | 571 (8.8%) |  | 260 (10.8%) | 233 (8.6%) | 78 (5.6%) |  |
|  | Asia and Ocean | 11481 (2.1%) | 181 (2.8%) |  | 96 (4.0%) | 65 (2.4%) | 20 (1.4%) |  |
|  | Middle east | 45507 (8.3%) | 915 (14.0%) |  | 377 (15.6%) | 373 (13.8%) | 165 (11.8%) |  |
|  | East Eu and Russia | 7394 (1.3%) | 68 (1.0%) |  | 26 (1.1%) | 24 (0.9%) | 18 (1.3%) |  |
|  | South America | 11520 (2.1%) | 212 (3.3%) |  | 69 (2.9%) | 92 (3.4%) | 51 (3.7%) |  |
|  | Unknown/Missing | 5739 (1.0%) | 86 (1.3%) |  | 41 (1.7%) | 35 (1.3%) | 10 (0.7%) |  |
| **Father's region of origin** | Sweden | 413612 (75.2%) | 4204 (64.5%) | <0.001 | 1439 (59.7%) | 1774 (65.5%) | 991 (71.0%) | <0.001 |
|  | Nordics | 19580 (3.6%) | 236 (3.6%) |  | 62 (2.6%) | 89 (3.3%) | 85 (6.1%) |  |
|  | West Eu and NA | 16143 (2.9%) | 197 (3.0%) |  | 87 (3.6%) | 63 (2.3%) | 47 (3.4%) |  |
|  | Africa | 21701 (3.9%) | 518 (7.9%) |  | 239 (9.9%) | 222 (8.2%) | 57 (4.1%) |  |
|  | Asia and Ocean | 17317 (3.1%) | 243 (3.7%) |  | 129 (5.4%) | 89 (3.3%) | 25 (1.8%) |  |
|  | Middle east | 39192 (7.1%) | 771 (11.8%) |  | 328 (13.6%) | 314 (11.6%) | 129 (9.2%) |  |
|  | East Eu and Russia | 11985 (2.2%) | 143 (2.2%) |  | 59 (2.4%) | 60 (2.2%) | 24 (1.7%) |  |
|  | South America | 10669 (1.9%) | 204 (3.1%) |  | 67 (2.8%) | 99 (3.7%) | 38 (2.7%) |  |
|  | Unknown/Missing | 17 (<1%) | 0 (0.0%) |  | 0 (0.0%) | 0 (0.0%) | 0 (0.0%) |  |
| **Maternal psychiatric diagnosis** | Not present | 311676 (56.6%) | 2919 (44.8%) | <0.001 | 1147 (47.6%) | 1228 (45.3%) | 544 (39.0%) | <0.001 |
|  | Present | 238540 (43.4%) | 3597 (55.2%) |  | 1263 (52.4%) | 1482 (54.7%) | 852 (61.0%) |  |
| **Paternal psychiatric diagnosis** | Not present | 399806 (72.7%) | 4220 (64.8%) | <0.001 | 1628 (67.6%) | 1751 (64.6%) | 841 (60.2%) | <0.001 |
|  | Present | 150410 (27.3%) | 2296 (35.2%) |  | 782 (32.4%) | 959 (35.4%) | 555 (39.8%) |  |
| **Maternal infection** | No | 219388 (39.9%) | 2289 (35.1%) | <0.001 | 725 (30.1%) | 977 (36.1%) | 587 (42.0%) | <0.001 |
|  | Yes | 330828 (60.1%) | 4227 (64.9%) |  | 1685 (69.9%) | 1733 (63.9%) | 809 (58.0%) |  |
| **Paternal infection** | No | 317992 (57.8%) | 3688 (56.6%) | 0.052 | 1306 (54.2%) | 1545 (57.0%) | 837 (60.0%) | 0.001 |
|  | Yes | 232224 (42.2%) | 2828 (43.4%) |  | 1104 (45.8%) | 1165 (43.0%) | 559 (40.0%) |  |
| **Family income at birth** | Low (0-20%) | 78377 (14.2%) | 1520 (23.3%) | <0.001 | 511 (21.2%) | 629 (23.2%) | 380 (27.2%) | <0.001 |
|  | Medium-low (20-40%) | 115721 (21.0%) | 1691 (26.0%) |  | 606 (25.1%) | 727 (26.8%) | 358 (25.6%) |  |
|  | Medium (40-60%) | 119491 (21.7%) | 1324 (20.3%) |  | 495 (20.5%) | 539 (19.9%) | 290 (20.8%) |  |
|  | Medium-high (60-80%) | 118690 (21.6%) | 1030 (15.8%) |  | 379 (15.7%) | 436 (16.1%) | 215 (15.4%) |  |
|  | High (80-100%) | 117294 (21.3%) | 859 (13.2%) |  | 355 (14.7%) | 358 (13.2%) | 146 (10.5%) |  |
|  | Missing | 643 (0.1%) | 92 (1.4%) |  | 64 (2.7%) | 21 (0.8%) | 7 (0.5%) |  |
| **Parental education** | <10 years | 33669 (6.1%) | 824 (12.6%) | <0.001 | 222 (9.2%) | 335 (12.4%) | 267 (19.1%) | <0.001 |
|  | 10-12 | 209705 (38.1%) | 3095 (47.5%) |  | 960 (39.8%) | 1364 (50.3%) | 771 (55.2%) |  |
|  | >12 | 305487 (55.5%) | 2501 (38.4%) |  | 1162 (48.2%) | 989 (36.5%) | 350 (25.1%) |  |
|  | Missing | 1355 (0.2%) | 96 (1.5%) |  | 66 (2.7%) | 22 (0.8%) | 8 (0.6%) |  |

Intellectual disability (ID), body mass index (BMI), Gestational age (GA), North America (NA), Europe (Eu)
